# PROmiRNA: a new miRNA promoter recognition method uncovers the complex regulation of intronic miRNAs

**DOI:** 10.1186/gb-2013-14-8-r84

**Published:** 2013-08-16

**Authors:** Annalisa Marsico, Matthew R Huska, Julia Lasserre, Haiyang Hu, Dubravka Vucicevic, Anne Musahl, Ulf Andersson Orom, Martin Vingron

**Affiliations:** 1 Max Planck Institute for Molecular Genetics, Ihnestrasse 63-73, 14195 Berlin, Germany; 2 Partner Institute for Computational Biology, Shanghai Institutes for Biological Sciences, Chinese Academy of Sciences, 320 Yue Yang Road, 200031 Shanghai, China

**Keywords:** miRNA transcriptional regulation, deepCAGE, mixture model

## Abstract

The regulation of intragenic miRNAs by their own intronic promoters is one of the open problems of miRNA biogenesis. Here, we describe PROmiRNA, a new approach for miRNA promoter annotation based on a semi-supervised statistical model trained on deepCAGE data and sequence features. We validate our results with existing annotation, PolII occupancy data and read coverage from RNA-seq data. Compared to previous methods PROmiRNA increases the detection rate of intronic promoters by 30%, allowing us to perform a large-scale analysis of their genomic features, as well as elucidate their contribution to tissue-specific regulation. PROmiRNA can be downloaded from http://promirna.molgen.mpg.de.

## Background

miRNAs are non-coding RNAs, approximately 22 nucleotides long, which have been shown to be crucial post-transcriptional regulators of gene expression in metazoans and plants, targeting up to 50% of the protein-coding genes [1,2]. Most of the research over the past decade has concentrated on elucidating the mechanisms of miRNA-mediated post-transcriptional regulation in cancer and other diseases, and on the potential clinical applications of this knowledge [1,3].

However, it is still poorly understood how miRNAs themselves are regulated. This is partly due to the difficulty of predicting promoters from short conserved sequence features without producing a high number of false positives [1,3-5], and partly due to the heterogeneity of the miRNA biogenesis pathways. Although in the past few years several promoter prediction methods have achieved very good performance when predicting gene promoters using a variety of machine learning techniques [6-8], there has been little progress in the field of miRNA promoters. miRNAs, whether they are located in intergenic regions or embedded within introns of protein-coding genes, are in most cases generated from long primary transcripts (pri-miRNAs). These transcripts, which can be up to several kilobases long, are then rapidly cleaved in the nucleus by the enzyme Drosha [3]. This presents a technical barrier for large-scale identification of miRNA transcription start sites (TSSs), as they can be located very far away from the mature miRNA and therefore cannot be easily inferred from the genomic location of the mature miRNA. The difficulty of experimentally detecting and consequently annotating miRNA promoters has limited our ability to identify the regulatory circuits that control miRNA expression, and has therefore prevented a comprehensive analysis of intronic miRNA promoter characteristics and usage easily inferred from the genomic location of the mature miRNA.

A few studies indicate that intronic miRNAs (that is miRNAs located inside the introns of other genes) are not necessarily co-transcribed with their host gene, which suggests that they might have their own independent intronic promoters [9,10]. The role of intronic miRNA promoters is largely unknown and this adds another layer of complexity to the transcriptional regulation of miRNAs. In addition, recent studies indicate that several alternative miRNA biogenesis pathways exist, for instance the one giving rise to splicing-derived miRNAs, called mirtrons [11]. Despite this complexity, miRNA TSS identification is a crucial step in understanding miRNA regulation, locating the core promoters, and searching for putative transcription factor binding sites (TFBSs).

Existing miRNA promoter recognition methods can be organized into two categories. Chromatin signature-based methods use histone mark profiles, such as H3K4me3 [12,13] or nucleosome positioning patterns [9] in specific cell lines to annotate miRNA promoters *de novo*. Supervised methods trained on protein-coding gene features exploit the evidence that miRNA promoters present the typical characteristics of promoters controlled by RNA Polymerase II [14] to build classification models from protein-coding gene promoters and apply them to distinguish miRNA promoters from non-promoters [10,15,16].

Although histone mark-based methods can identify up to 80% of miRNA promoters, they have been designed for specific cell lines [12,13]. Additionally, due to the nature of ChIP-seq experiments and the nucleosomes themselves, histone mark profiles provide a broader view of gene promoters, rather than a direct readout of promoter activity and usage. As a consequence, chromatin-based methods represent a valuable strategy for detecting intergenic and host gene miRNA promoters, but they might lack the sensitivity required to identify intronic promoters. Concerning the second category of methods, we believe that, although miRNA and protein-coding gene promoters show several similarities, this is mainly true for intergenic miRNAs, as too little is still known about intronic miRNA promoters. For this reason, a supervised method trained on protein-coding genes might not be the optimal choice for identifying miRNA promoters, especially intronic ones. On the other hand, given the limited number of known miRNA TSSs, it does not seem feasible to build a supervised model using miRNA promoter annotation only.

In this study we present a novel methodology for annotating miRNA promoters called PROmiRNA. Our model is a semi-supervised classification mixture model and uses the deepCAGE data from several tissues, which was generated by the FANTOM4 project [17], together with sequence features, to distinguish putative miRNA promoters from background noise. We apply PROmiRNA to the human genome, to annotate all alternative miRNA promoters and analyze their regulatory features, especially those that characterize intronic promoters. The semi-supervised approach ensures that a minimal number of initial assumptions are made about the nature of miRNA promoters and their similarities to protein-coding gene promoters, while the use of several deepCAGE libraries ensures a high coverage of the method, allowing us to identify at least one TSS for about 82% of the miRNAs annotated in miRBase [18]. We compare PROmiRNA to the methods from Barski *et al. *[12] and Ozsolak *et al. *[9] and we find that up to 82% of the miRNA promoters annotated by these two studies are also discovered by PROmiRNA. In addition, PROmiRNA returns all possible alternative promoters for each miRNA, which are mostly missed by the other two methods. We also found that up to 62% of annotated intragenic miRNAs have their own promoters, independent of the host gene promoter, compared to 30% to 35% reported from previous studies [9,10]. In the absence of a large set of known miRNA TSSs against which to validate our novel promoters, we compute an indirect measure of the performance of our method based on the overlap of the identified promoters with PolII ChIP-seq data from the ENCODE project. We obtain a precision of 83% and 76% for intergenic and intronic miRNA promoters, respectively. We also find that up to 85% of our newly annotated promoters are significantly enriched in read coverage from RNA-seq experiment data, evidence that supports our predictions. Additionally, we validate the promoters identified by our model with a significant number of existing annotated miRNA TSSs and we are able to experimentally validate the identified promoter for two miRNAs, one of which is a novel intronic promoter for miR-130a which has not previously been described.

Recent studies of mammalian promoters suggest that alternative promoters of non-coding transcripts are often located inside introns and can be associated with various disorders including cancer [19]. This might also be the case for intronic miRNA promoters and raises important questions about their functional role, their contribution to miRNA expression versus host gene promoters and their evolution. To the best of our knowledge, these questions remain unanswered and the characteristics of intronic promoters, as well as their mechanisms of regulation and evolution have not been systematically investigated.

In this study we attempt to answer these questions and find that intronic promoters convey an additional degree of freedom over intragenic miRNA transcriptional regulation, allowing miRNA expression levels to be modulated in a tissue- and condition-specific manner. We discuss the regulatory features of intronic miRNA promoters, compared to other miRNA promoter classes, and we propose for the first time a model for intronic promoter usage and evolution. PROmiRNA can be used to identify and study miRNA promoters in any other species where either deepCAGE or TSS-seq data are available and can be applied to already annotated mature miRNAs, as well as newly discovered miRNA from sequencing experiments. The source code for PROmiRNA, as well the original data used in this study are free for download [20].

## Results

### Identification of candidate miRNA promoter regions and statistical modeling

In this study we propose a semi-supervised mixture model for miRNA promoter recognition, which uses the deepCAGE data generated within the FANTOM4 Consortium as well as sequence features in order to separate putative promoters from background noise.

**Figure 1:**
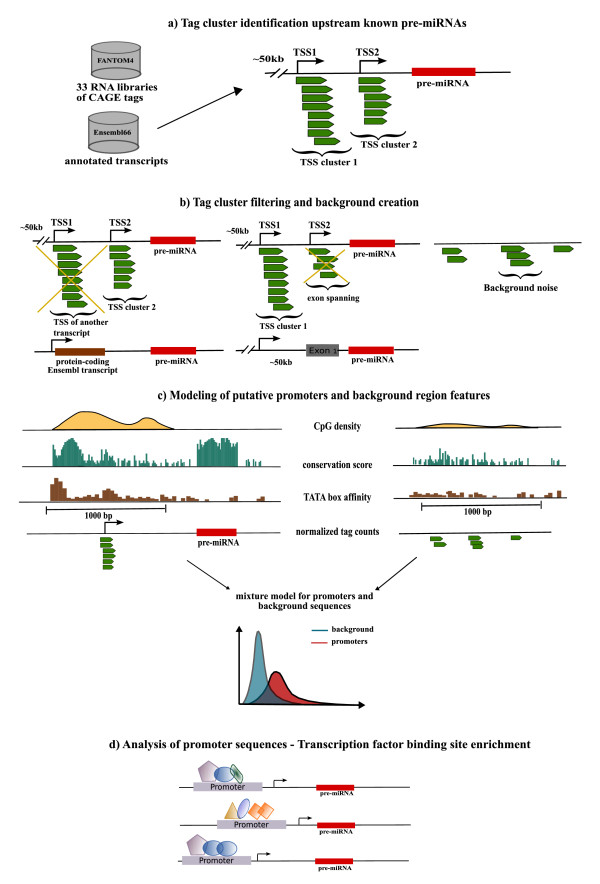
**Workflow of the algorithm and analysis**. **(a) **DeepCAGE data from 33 FANTOM4 RNA libraries are used to define transcription start site (TSS) clusters of overlapping tags in regions up to 50 kb upstream of annotated precursor miRNAs (pre-miRNAs). **(b) **TSS clusters overlapping with the gene starts of other annotated Ensembl transcripts are filtered out, as well as tags spanning exonic regions. **(c) **Sequence features, such as CpG content, conservation score and TATA box affinity, are calculated in both 1,000 bp long regions around putative TSSs and in random intergenic regions, and, together with read count distributions, are used to model the mixture of the two promoter and non-promoter classes. **(d) **Those regions whose probability of being a promoter is higher than the probability of being noise are used to analyze general characteristics of miRNA promoters, such as enrichment of specific transcription factor binding sites. bp, base pair; kb, kilobase; pre-miRNA, precursor miRNA; TSS, transcription start site.

Genomic regions enriched in cap analysis of gene expression (CAGE) tags presumably correspond to transcript TSSs, therefore our algorithm starts with scanning the genome in the regions up to 50 kb upstream of an annotated miRNA precursor, searching for clusters of mapped CAGE tags (Figure 1). Such regions represent an initial set of candidate miRNA promoters. A probabilistic model is trained on a data set composed of tag counts in the candidate human miRNA promoters (the unlabeled set) and in randomly selected intergenic and intronic background regions (the negative set), as described in Materials and methods. When plotting the read count distributions from the pooled FANTOM4 libraries, one observes an approximately bimodal behavior (see Figure S3a in Additional file 1) and it is tempting to assume that the distribution of the data is represented by a mixture model of putative promoters versus background noise. However, while some miRNA promoters are expressed at higher levels, that is a high number of read counts, some lowly expressed regions might fall into the distribution of the noise. For this reason, in addition to tag counts, other promoter features, such as CpG content, conservation score, TATA box affinity and mature miRNA proximity, are introduced into the model through an informative prior probability density function (see Materials and methods).

The optimal separation between the promoter class and the background noise class is learned directly from the data described above, including both CAGE tags and promoter features in the learning process. After training, the classifier is able to give the posterior probability that a given region is a miRNA promoter. The regions with a posterior probability higher than 0.5 (that is, they are more likely to be a promoter than not), are labeled as such and assigned to the miRNAs downstream within 50 kb.

The application of our algorithm to the human genome allowed us to generate a reliable set of miRNA promoters. This set was used to perform a detailed analysis of promoter features in different miRNA classes and to interpret them in terms of promoter usage, miRNA expression and miRNA evolution.

### High-throughput miRNA promoter discovery

Annotated human miRNAs were extracted from miRBase v18. Table 1 gives the number of identified promoters and the number of miRNAs that could be assigned to at least one promoter for five promoter categories: all, intergenic, host gene, intronic and hybrid promoters. The miRNAs were classified as intergenic (if they were located in regions between annotated genes) or intragenic (if they overlapped with exons or introns of other genes on the same strand). Genes that contained a miRNA inside one of their introns were referred to as host genes. Hybrid promoters are defined as those promoters which fall in intergenic regions upstream of intragenic miRNAs and could not be assigned unambiguously to the miRNA. From 1,506 human miRNAs contained in miRBase, 465 were classified as intergenic and 1,041 as intragenic. After running PROmiRNA on these miRNAs, 1,228 (82.6%) could be associated with at least one identified TSS (see Table 1). A promoter could not be identified for the remaining 218 miRNAs (17.4%). This could be due to several reasons: the miRNA might not be expressed in the analyzed cell lines or tissues, the miRNA might be transcribed by RNA Polymerase III and therefore not detectable by means of deepCAGE, the miRNA-associated promoter could be located further than 50 kb upstream of the annotated precursor and therefore not detectable by our algorithm or the miRNA-associated primary transcript might be degraded too quickly to be detected by cap trapping. For about half of the miRNAs without identified promoters we found promoter-like regions upstream of the mature miRNA, which we referred to as hybrid because they could not be unambiguously assigned to miRNAs. We classified our identified TSSs as intergenic (if they belonged to intergenic miRNAs), intronic (if they were located inside annotated introns of genes hosting intragenic miRNA) or as a host gene (if they overlapped with a genomic region of 200 bp around the annotated host gene's TSS). For approximately 80% of the intragenic miRNAs we identified the promoter of the associated host gene, their own intronic promoter or both. In total, 60.5% of the intragenic miRNAs could be associated with an independent intronic promoter and 60% to their host gene promoter (see Table 1).

**Table 1:** Results from PROmiRNA on human miRNAs

Category	All	Intergenic	Host gene	Intronic	Hybrid
Number of promoters	7,244	1,386	1,815	2,037	2,006
Percentage of miRNAs with at least one promoter	82.6%	83.7%	60%	60.5%	-
Percentage of miRNAs with more than one promoter	84%	80%	69%	74%	-

We identified a promoter for 389 out of the 465 intergenic miRNAs (83.7%, Table 1). The algorithm assigned on average 4.7 alternative TSSs per miRNA, and 81% of the miRNAs had more than one identified TSS (see Table 1). This evidence indicates that alternative promoters are a common mechanism for creating diversity in miRNA transcriptional regulation, as previously observed for protein-coding genes [21].

A minor sub-class of intragenic miRNAs exists, which includes exonic miRNAs, that is miRNAs that reside in exons of transcripts, mainly non-coding transcripts [22]. The number of exonic miRNAs annotated in mirBase is small (89) compared to the majority of intronic miRNAs. We analyzed the exonic miRNAs separately and observed that most of them (62%) do not have a predicted intronic promoter, indicating that they might be products of splicing from host-gene transcripts. For the remaining 34 exonic miRNAs the predicted intronic promoters most likely correspond to alternative TSSs of the non-coding transcripts that contain them and they might drive the expression of such miRNAs independently.

Likewise, several hundred novel mirtrons, that is spliced intronic miRNAs, have been recently discovered in human and mouse [11]. Out of 51 mirtron-like miRNAs, re-annotated in mirBase from Ladewig *et al*., 34 are predicted also to have independent intronic promoters, most in a different intron than the one containing the miRNA. Transcription of intronic miRNAs driven by a promoter located in another intron of the same transcript has been postulated in the past and also experimentally validated in a few cases [9]. The evidence that mirtrons can also have an independent promoter points to the possibility of a competing process, or more generally cross-talk, between the splicesome and the miRNA processing complex [23]. However, only experimental validation could confirm whether this is actually the case.

The complete list of all identified miRNA promoters, together with their genomic coordinates, features and prior and posterior probabilities, is provided in Additional file 2.

### Comparison with existing annotation and other miRNA promoter recognition methods

#### miRNAs TSSs annotated in the literature

To assess the reliability of our method we checked whether or not we were able to re-annotate known miRNA TSSs that have been described in the scientific literature, as well as miRNA TSSs that were recognized using other methods. Our method could correctly identify the transcription start site of miR-122, located 4,800 bp upstream of the precursor miRNA (pre-miRNA). This TSS was validated by Chien *et al*. with the presence of ESTs in the TSS proximity, starting at the promoter and spanning the mature miRNA region, and experimentally proven using a promoter reporter assay [16]. We also detected the conserved promoter of miR-146a in the lung and monocytic cell line RNA libraries, located about 17 kb upstream of the precursor miRNA, as well as the miR-146b promoter, 40 kb away from its miRNA precursor [24]. We could correctly identify the promoter of miR-155, mainly in the RNA libraries from the monocytic and immune system cell lines, as coincident with the promoter of its host gene [25]. We could also correctly identify the high-CpG promoter of the intergenic miR-34a, about 30 kb upstream of the precursor miRNA [26], and the conserved TATA-box main promoter of miR-21 [27], as well as closer alternative promoters, like the one located about 2,000 bp upstream of the precursor, already discovered by Ozsolak *et al*. in a nucleosome-depleted region [9]. Finally, we were able to detect the TSS of miR-663a, about 180 bp upstream of the miRNA precursor, and the TSS of miR-17, 2,220 bp upstream of the precursor, both annotated in Ensembl. We could not verify the transcription start region of miR-127 located a few hundred base pairs upstream of the pre-miRNA, which was found to be induced in a urinary bladder carcinoma cell line and in fibroblasts, independently of the other members of the same miRNA cluster (miR-136, miR-431, miR-432 and miR-433) [28]. This is possibly due to the fact that the cell lines investigated in [28] were not present in the RNA libraries used for this study.

#### Comparison with histone mark-based methods

We chose two recent miRNA promoter annotation methods for comparison with our method, the one from Barski *et al*., which uses histone modification data, such as H3K4me3 and H2A.Z profiles [12], and the other one from Ozsolak *et al*., which mainly uses chromatin structure to define high-scoring putative miRNA promoters in nucleosome-free regions [9]. This choice is motivated by two reasons: first, these two approaches are *de novo *miRNA promoter discovery methods; second, the studies from Zhou *et al. *[15] and Chien *et al. *[16], based on supervised classification models, either intentionally exclude intronic miRNAs from their analysis or do not report a list of intronic miRNA promoters, preventing a comparison for this specific promoter class.

The number of miRNAs that could be assigned to at least one promoter is reported in Table 2 for PROmiRNA and the other two methods, the one from Barski *et al. *[12] and the one from Ozsolak *et al. *[9]. miRNAs promoters fall into four categories: all promoters, intergenic, intragenic and intronic promoters. Intragenic promoters are promoters assigned to intragenic miRNAs and include both host gene promoters and intronic promoters.

**Table 2:** Comparison of PROmiRNA with other methods

Percentage of miRNAs with a promoter	PROmiRNA	**Barski *et al***.	**Ozsolak *et al***.
All	82.6% (1,228/1,506)	43% (233/541)	48% (177/370)
Intergenic	83.7% (389/465)	28% (50/182)	38.4% (40/104)
Intragenic	80% (831/1,041)	50% (172/343)	65.2% (137/210)
Intronic	60.5% (630/1,041)	14% (47/343)	31% (43/210)

A strict comparison with the results from these two studies is hard due to the fact that they used specific cell lines, CD4+ cells in [12] and MCF7 and MALME in [9], respectively, to investigate miRNA promoters, and they could assign a promoter to at most 48% of the analyzed miRNAs (probably corresponding to miRNAs actively transcribed in those cells). In contrast, we used 33 RNA libraries, corresponding to different tissues and cell lines, which ensures a higher coverage. In addition, the miRBase annotation changes quickly as more and more miRNAs are discovered: while Barski and Ozsolak analyzed 541 and 370 miRNAs, respectively, we annotated TSSs for about 1,500 known miRNAs. Nonetheless, a rough comparison was possible: we checked for overlaps between genomic regions within 500 bp of our identified TSSs and the promoter regions reported by the other two methods.

In the comparison with Barski's method [12], we found overlapping annotations for 110 out of 177 (62%) of their promoters. Of the remaining 67 miRNA promoters that were recognized by Barski but not by our method, we investigated the distances of the promoters from their pre-miRNAs and found that 57 of their promoters were located more than 50 kb upstream of the annotated pre-miRNA, in regions that were not scanned by our method. When we extended our algorithm to scan genomic regions beyond 50 kb we found an overlap for 35 more TSSs, for a total of 82% common TSSs between our method and Barski's method. Similarly, in the comparison with Ozsolak's method [9], we found that our annotated TSSs overlapped 153 out of 188 (81.4%) of the TSSs found by Ozsolak.

On top of identifying most of the miRNA promoters identified by the other methods, PROmiRNA also returned all possible alternative promoters for a certain miRNA, including the intronic promoters. We divided the annotated promoters from the previous two methods into intronic and host gene promoters, based on miRBase v18 and Ensembl v66 annotation and observed that PROmiRNA had a substantially increased detection rate for intronic miRNA promoters (see Table 2): it could assign an intronic promoter to about 62% of the intragenic miRNAs, versus 14% in [12] and 35% in [9]. This sensitivity for intronic miRNA promoters is, to the best of our knowledge, unique to our method and has allowed us to study the properties of intronic promoters for the first time.

#### Ability of PROmiRNA to identify miRNA promoters at high resolution

**Figure 2:**
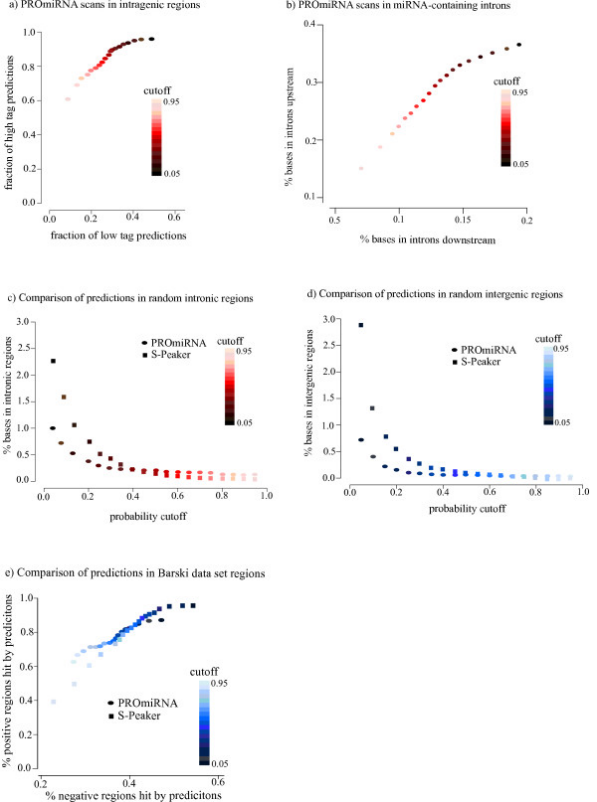
**Performance of PROmiRNA on intronic and intergenic data set**. **(a) **The fraction of predicted highly expressed CAGE tag clusters versus lowly expressed tag clusters in intronic miRNA regions at varying probability cutoffs. **(b) **The percentage of PROmiRNA predictions in intronic regions upstream of annotated miRNAs (upstream regions) versus the percentage of predictions in intronic regions downstream of miRNAs (downstream regions) at different probability cutoffs. **(c) **The percentage of bases covered by predictions in random intronic regions versus the probability threshold for both PROmiRNA and S-Peaker. **(d) **The percentage of bases covered by predictions in random intergenic regions versus the probability threshold for both PROmiRNA and S-Peaker. **(e) **The percentage of Barski miRNA promoters (positive regions) versus the percentage of negative regions hit by predictions at different probability cutoffs for both PROmiRNA and S-Peaker.

To get a better understanding of how our model behaves on both intergenic and intragenic regions we performed several controls. To prove that PROmiRNA is effective in predicting highly expressed intronic promoters versus additional intronic regions, we extracted a held-out test set of 5,000 intronic regions upstream of annotated miRNAs, where CAGE tags were present. In the absence of true labels for intronic promoters, highly expressed tag regions have a higher probability of representing true promoters. We therefore defined highly expressed CAGE tag regions as those tag clusters with a quantile-normalized number of tags of at least 3.0. Figure 2a shows the fraction of highly expressed CAGE-tag intronic regions versus the fraction of additional predicted intronic regions for probability cutoffs ranging from 0.05 to 0.95. We observe a significantly higher proportion of highly expressed tag regions compared to lowly expressed tag regions (3.3 fold change) at a probability cutoff of 0.5, a level where 88% of the highly expressed tag regions are retrieved by PROmiRNA. This fold change increases further at higher probability thresholds.

Figure 2a indicates that PROmiRNA picks up high-tag miRNA intronic promoters at high resolution, but we also observe a considerable number of low tag predictions at all the probability cutoffs. These additional regions may correspond, to a large extent, to lowly expressed miRNA promoters that PROmiRNA predicts when the prior probability has a stronger impact on the final probability than the tag counts.

To demonstrate the ability of PROmiRNA to correctly recover intronic promoters, we extracted two sets of regions: an upstream set containing intronic regions upstream of each intronic miRNA and a downstream set containing intronic regions downstream of each intronic miRNA. For simplicity, we limited this analysis only to the miRNA-containing introns, although transcription of intronic miRNAs from TSSs located in different introns has also been observed. We ran PROmiRNA on both the upstream and downstream regions and computed the percentage of bases covered by predictions in both sets at different probability cutoffs. As shown in Figure 2b, at a probability threshold of 0.5 the percentage of bases covered by PROmiRNA predictions is significantly higher (about a threefold change) in the upstream set than in the downstream set. Although we cannot exclude the possibility that a fraction of the predicted TSSs downstream of an intronic miRNA correspond to true transcript starts, the enrichment in predicted TSSs upstream of intronic miRNAs supports our hypothesis that these miRNAs are transcribed from their own intronic promoters.

To assess how PROmiRNA behaves on randomly selected regions we computed the percentage of bases covered by PROmiRNA predictions on a set of 5,000 random intergenic regions as well a set of 5,000 random intronic regions, all of which did not contain a miRNA, at different probability cutoffs. We compared our results to the predictions obtained by scanning the same sets of regions with S-Peaker [8], a highly precise TSS prediction method for gene promoters in general, which is based on enrichment of known transcription factor signals. The comparison between the two tools is not straightforward because S-Peaker returns a per-base probability for each input region while PROmiRNA outputs a global posterior probability for the same region. Nevertheless, the fraction of bases covered by predictions should represent a fair measure of comparison. Figures 2c and 2d show that for a probability cutoff equal to or higher than 0.5 the fraction of bases covered by PROmiRNA predictions is very low and highly comparable to the fraction obtained with S-Peaker. This indicates that the predictions in both random intergenic and intronic regions may include some real TSSs that have not yet been annotated.

Finally, we investigated the ability of PROmiRNA to retrieve the miRNA promoter regions from Barski *et al. *[12] at high recall. To do this, from the 541 human miRNA promoter regions in Barski we retained a set of 114 unique regions after removing the predicted regions located more than 50 kb upstream of the mature miRNA and collapsing the promoter annotation for miRNAs in the same cluster. In addition, we kept only the most upstream miRNA of each cluster. These selected regions mainly correspond to intergenic miRNA promoter regions, as shown in the previous paragraph. We defined a set of positive regions - the 114 annotated miRNA promoters from Barski - and a set of negative regions comprising those sequences between the miRNA promoter and the miRNA location itself. We scanned both positive and negative regions with PROmiRNA and compared the percentage of positive regions versus the percentage of negative regions hit by our predictions at different probability cutoffs. We scanned the same regions with S-Peaker and the results are summarized in Figure 2e. We observe a higher percentage of positive regions hit by PROmiRNA predictions (2.3 fold change) compared to the negative regions at a probability threshold of 0.5, where about 77% of the positive regions contain at least one PROmiRNA prediction. The results are comparable with S-Peaker at this cutoff. For higher cutoffs we observe a greater than threefold change for both PROmiRNA and S-Peaker. These results again show that our predictions correlate well with Barski annotation. While the PROmiRNA predictions are enriched in the positive regions, we cannot exclude the possibility that the predictions in negative regions correspond to alternative TSSs active in some of the 33 FANTOM cell lines but not detectable from Barski's method, which only looks at histone mark signals.

### Validation of our approach on PolII data and RNA-seq data

In the absence of a sufficiently large benchmark set of miRNA promoters it is difficult to know whether the identified promoters, including the intronic miRNA promoters that are uniquely recognized by our method, correspond to true promoters. We therefore decided to develop two indirect means of evaluating the precision of our approach, by integrating information from two different types of genomic data. First, we validated the positions of our identified promoters with the PolII ChIP-seq annotation from all libraries in the ENCODE project (HAIB track in Additional file 1) and second, we validated the existence of full-length pri-miRNAs generated at the identified promoters by means of the RNA-seq data from the Human Body Map 2.0 Project [29]. In addition, we experimentally assessed the promoter activity of two new annotated promoter regions, for miR-122 and miR-130a, by means of a promoter reporter assay.

#### PolII binding supports the majority of newly annotated miRNA promoters

**Figure 3:**
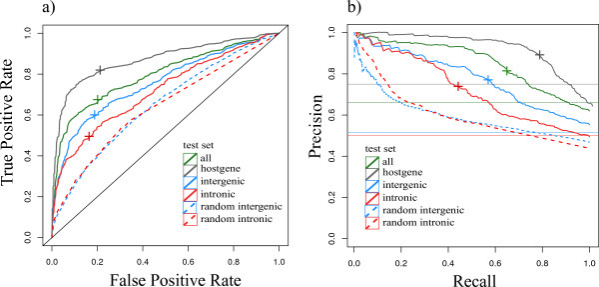
**Performance of PROmiRNA based on a comparison with PolII ChIP-seq data from the ENCODE project**. **(a) **ROC computed by varying the posterior probability cutoff for four promoter classes and two sets of random intergenic and intronic regions. The '+' symbols correspond to the recall values and false positive rates at the decision cutoff of our binary classifier, *p*(prom) = 0.5 for the four promoter classes. **(b) **Precision-recall curve computed by varying the posterior probability cutoff *c *for four promoter classes and two sets of random intergenic and intronic regions. The horizontal lines correspond to the random case. The '+' symbols correspond to the recall and precision values calculated at a cutoff *c *= 0.5, which represents the minimum required posterior probability *p*(prom) from our classifier to classify candidate regions as belonging to the promoter class. The precision of the method in identifying promoters at a cutoff of *p*(prom) = *c *= 0.5 is equal to 83%, 92%, 78% and 75% for all, host gene, intergenic and intronic promoters, respectively. ROC, receiver operating.

To evaluate our method we performed cross-validation five times, each time using a random set of 8,000 candidate regions and compared the predicted promoters with pre-computed PolII peaks pooled from all the ChIP-seq experiments in the HAIB TFBS ENCODE project track. An identified promoter overlapping an annotated PolII peak was counted as a true positive, while an identified promoter that did not overlap any annotated PolII peak was counted as a false positive. By varying the promoter posterior probability cutoff we built a precision-recall curve (Figure 3b) and a ROC (Figure 3a) for all regions in the test sets, as well as for intergenic, intronic and host gene putative promoters separately, as well as for two sets of random intergenic and intronic regions. A cutoff of 0.5 for the posterior promoter probability corresponds to the situation in which 81%, 60% and 49% of the candidate regions are classified into the promoter class (*p*(prom) > 0.5) at a precision of 92%, 78% and 75% for host gene, intergenic and intronic promoters, respectively. Also, at a cutoff of 0.5 the false positive rate of the method is less than 20% for all promoter classes (Figure 3a). The numbers reported in Table 1, as well as the regions in Additional file 2, correspond to the predicted miRNA promoters at a cutoff of 0.5 and at a false positive rate of less than 20%, according to PolII annotation. Figure 3 also shows that the false positive rate for both random intergenic and intronic regions is worse than for intergenic and intronic regions upstream of annotated miRNAs, but still better than random. This can be explained by the fact that there is pervasive transcription in intergenic regions corresponding to transcription start sites of unannotated transcripts, especially non-coding RNAs. Similarly, the method might predict TSSs in random intronic regions that correspond to alternative TSSs or intronic promoters of the corresponding transcripts.

Based on the PolII comparison, we also show (Figure S2 in Additional file 1) that the model used in this study, which combines CAGE tag counts and promoter features, outperforms both a simple model, which only uses CAGE tag counts, as well as a model that only uses the information from the prior probability, that is promoter features, on the same set of test regions.

In light of the fact that comparison with PolII peaks is an indirect way to validate our transcription start sites, one would expect a certain amount of disagreement. Therefore, the false positive rate of 18% when comparing our predictions to PolII peaks is indicative of the very good performance of the method.

#### Most of the identified miRNA transcription start sites are validated by full-length RNA-seq coverage data

Assuming that our identified TSSs are correct, we would expect to find full-length primary transcripts that originate at an identified TSS and continue through the annotated mature miRNA. To investigate this, we used RNA-seq data from the Human Body Map 2.0 Project from 16 different human tissues [29] and quantified the coverage of mapped reads in the regions spanning the identified pri-miRNAs. The rationale behind this approach is that one would expect to see an enrichment of read coverage in true putative primary miRNA transcripts compared to random genomic regions, where a putative pri-miRNA transcript is defined as that region starting at the predicted TSS and terminating at the end of the annotated miRNA precursor. Previous methods have used EST libraries to validate their predictions and check to which extent identified TSSs correspond to primary transcripts [9,12,13]. Here we took advantage of the fact that the RNA-seq data from the Human Body Map 2.0 Project was generated from pooled RNA extracted from 16 tissues, which largely overlap with the tissues used in the FANTOM4 project. Given the higher sequencing depth of such data compared to ESTs libraries, we are also able to detect lowly expressed transcripts.

**Figure 4:**
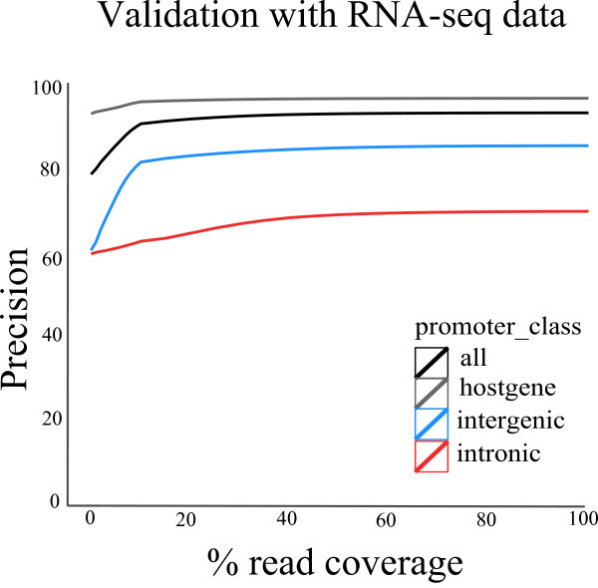
**Validation of PROmiRNA based on a comparison with RNA-seq data from the Human Body Map 2.0 Project**. The precision of the method, indirectly computed on the basis of RNA-seq data, is plotted against the percentage of continuously mapped reads in the identified pri-miRNA regions for four promoter classes.

The read coverage of regions between a TSS and the end of the mature miRNA and the significance of this coverage were calculated as described in Materials and methods. Primary transcripts showing significant read coverage were counted as present, while transcripts with a non significant coverage were considered as absent. We looked at the dependency of the computed precision (see Materials and methods) on the read coverage for host gene, intronic and intergenic primary transcripts (Figure 4) and we observed that, already at low coverage, the method performed very well at identifying host gene and intergenic transcripts (precision approximately 99% and 82% for host gene and intergenic transcripts, respectively), but was only able to achieve a precision of 65% for intronic transcripts. The lower precision for intronic transcripts could be due to the high number of RNA-seq reads mapping unspecifically in intronic regions (data not shown), which led to an overestimation of the coverage in the intronic background set and consequently an underestimation of the true number of intronic pri-miRNAs.

Precision values calculated on the basis of PolII data or RNA-seq data have to be interpreted as a lower bound for the following reasons: first, the cell lines inside the ENCODE project from where the PolII ChIP-seq data were generated are different from the libraries in FANTOM4, from where our annotations are derived. If the intronic promoters correspond to alternative promoters used in a tissue-specific manner, this could explain why a smaller number of them, compared to the intergenic promoter, were found to overlap a PolII peak. Second, pri-miRNAs that are rapidly degraded will show lower read coverage values, leading to an underestimation of the true positives using the RNA-seq coverage. Third, the high number of mapped reads in the introns might mask read-enriched regions corresponding to real intronic pri-miRNA transcripts; fourth, there is no way to discriminate real intronic pri-miRNA transcripts from unannotated alternative transcripts of the host gene. Overall, the results indicate that our method can be considered highly reliable in identifying both intergenic and intronic promoters but that evaluating intronic miRNA primary transcripts is much more difficult. The promoters that could be validated by means of PolII or RNA-seq data, as well as a comparison with the other methods described above, are listed in Additional File 3.

#### Experimental validation of miR-122 and miR-130a promoters

**Figure 5:**
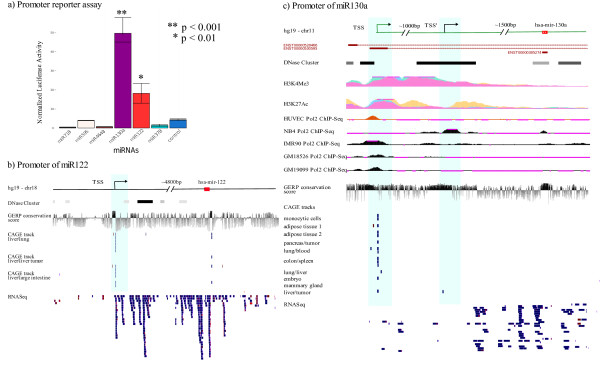
**Promoter reporter assay result and two validated miRNA promoters**. **(a) **Luciferase assay results on six identified miRNA promoters plus a control. The *y*-axis is the ratio between firefly luciferase and *Renilla reniformis *luciferase activity. Transfection efficiencies are normalized to the expression of a co-transfecting plasmid expressing *Renilla reniformis *luciferase. Experiments are shown as average plus/minus standard deviation of three independent transfections of three independent experiments. The promoter of miR-122 shows significantly higher activity with respect to the control (fold change approximately 5, *P *< 0.01). The miR-130a intronic promoter shows an activity about tenfold higher than the control (*P *< 0.0001). **(b) **Genomic characteristics of the region up to 5,000 bp upstream of pre-miR-122, including the validated promoter location (CAGE tracks), the RNA-seq read coverage and the conservation score from the UCSC Genome Browser. **(c) **Genomic characteristics of the 2,500 bp long region upstream of pre-miR-130a including the annotated transcripts of the host gene, the DNase clusters corresponding to open chromatin regions, the H3K4me3 and H3K27Ac histone marks (generally associated with active promoters), the PolII ChIP-seq peaks in different cell lines, the conservation score, the location of both host gene and intronic promoters (CAGE tracks) and the RNA-seq reads. The presence of a DNase cluster, the H3K4me3 and H3K27Ac marks and a second PolII peak in NB4 cells in the region of the identified intronic promoter, are additional evidence for the miR-130a intronic promoter.

To assess the real promoter activity of identified miRNA TSSs, five novel intronic miRNA promoters (miR-718, miR-130a, miR-595, miR-4648 and miR-378g) and one intergenic promoter (miR-122) were selected for experimental validation by means of a promoter reporter assay. The region from 500 bp upstream to 50 bp downstream of each identified TSS was cloned into a promoter-less plasmid of firefly luciferase. HEK293 cells were transfected with the constructs as described in Materials and methods. Cells transfected with promoter-less plasmids were used as controls. We verified the TSS of miR-122 located around 4,800 bp upstream of the annotated pre-miRNA (see Figure 5a,b), in agreement with the result from [16]. We also detected a highly significant increase in luciferase activity for the construct containing the intronic miR-130a identified promoter region, located about 1,600 bp upstream of the precursor (Figures 5a,c). Interestingly, miR-130a is located in the first intron of a long non-coding RNA (Ensembl id: ENSG00000254602). The host gene promoter is located about 2,500 bps from the precursor and is expressed in several RNA libraries, while the intronic promoter, whose evidence was supported also by PolII ChIP-seq data (Figure 5c), was specific to one library. The promoters of the other tested miRNAs could not be validated and this was probably due to the fact that such alternative promoters were tissue specific (miR-718 is T-cell specific and miR-595, miR-4648 and miR-378g are brain specific) and were not able to induce transcription in HEK293 cells.

### Genomic features of miRNA promoters

**Figure 6:**
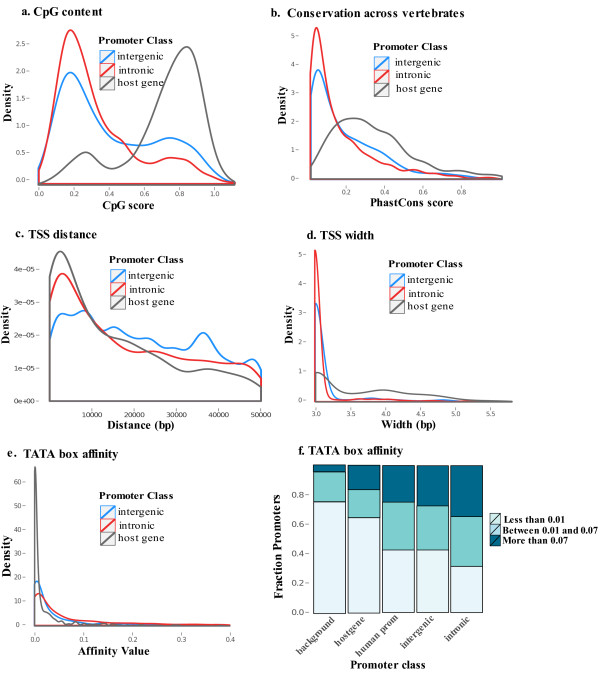
**Distribution of promoter features for intergenic, intronic and host gene promoters**. **(a) **Probability density function (PDF) of the CpG score across intergenic (blue), intronic (red) and host gene (gray) promoters. **(b) **PDF of the conservation score, calculated from the PhastCons vertebrate UCSC track, across intergenic, intronic and host gene promoters. **(c) **PDF of the distance between intergenic, intronic and host gene TSSs from miRNA precursors. **(d) **PDF of transcription start site (TSS) width, shown as log_10_(*width*) for clarity, for intergenic, intronic and host gene promoters. **(e) **PDF of the TATA box affinity values, calculated using TRAP, for intergenic, intronic and host gene promoters. **(f) **Bar plot showing the fraction of promoters that have a TATA box affinity lower than 0.01, in the range between 0.01 and 0.07 or higher than 0.07 for intergenic, intronic and host gene promoters, as well as for all human promoters and background regions. The two affinity levels of 0.01 and 0.07 correspond to the median and upper quantile affinity values for human promoters, respectively. bp, base pair; miRNA, microRNA; PDF, probability density function; TSS, transcription start site.

We identified 7,244 miRNA TSSs at a posterior promoter probability threshold of 0.5. We further analyzed the surrounding 1,000 bp regions of these high-confidence predictions. The predicted TSSs were divided into intergenic, intronic and host gene as described above. Our analysis showed that the three classes of promoters had different genomic features when considering CpG content, conservation across vertebrates, TATA box binding motifs, location with respect to the pre-miRNA and TSS width (Figure 6). Each of the features is discussed in detail below.

#### Distinct miRNA promoter classes differ in CpG content

CpG islands are known to co-localize with transcription start regions and are often used for promoter recognition. It is known that human promoters can be naturally divided into two classes according to whether they have low or high CpG content [30]. We determined the normalized CpG score for all promoters in the three miRNA promoter classes as described in Materials and methods, and looked at their distributions (Figure 6a). All three classes of promoters showed the separation into high CpG and low CpG promoters; however, the different promoter classes had substantially different CpG score distributions: the host gene promoters were mainly high CpG, while intergenic and especially intronic promoters were mostly low CpG.

#### Intronic miRNA promoters are less evolutionarily conserved than intergenic and host gene promoters

Previous studies showed that some miRNA promoters have a high level of conservation across vertebrates [31]. We found that the distributions of PhastCons conservation scores for intronic and intergenic promoters were significantly different from the host gene promoter distribution (Figure 6b), highlighting the fact that intronic promoters are less conserved than either host gene or intergenic promoters (*P *= 1.4 × 10^-92 ^and *P *= 7.8 × 10^-5^, respectively, Mann-Whitney U test).

#### miRNA transcription start sites are frequently located several kilobases upstream of the annotated precursor miRNAs

The majority of TSSs occurred within 10 kb upstream of the annotated pre-miRNA for intragenic miRNAs, while the TSS distances for intergenic miRNAs were significantly higher (*P *= 1.5 × 10^9 ^and *P *= 1.8 × 10^-7 ^for host gene and intronic TSSs, respectively). The distribution of the identified TSS distances from the pre-miRNAs for the three promoter classes is shown in Figure 6c.

#### Wide miRNA promoters are associated with CpG islands

Carnici *et al*. observed that the shape of human promoters is highly variable, ranging from very narrow promoters of 20 bp to 30 bp, characterized by a single peak of CAGE tags, to promoters up to several hundreds or even thousands of base pairs long, characterized by a broad distribution of CAGE tags [21]. The authors found a significant association between broad promoters and high CpG content, and narrow-peaked promoters and the presence of a TATA box binding site. We looked at the distribution of the logarithm of the promoter width for the three different miRNA promoter classes (Figure 6d). We observed that most of the intergenic and intronic miRNA promoters were narrow (less than 100 bp wide) compared to the large host gene promoters. Specifically, the percentage of promoters wider than 100 bp was 25% for the host gene promoters and 5% and 1% for intergenic and intronic promoters, respectively. We also confirmed the association observed by Carnici *et al*. between large promoters and high CpG promoters (*P *= 2.15 × 10^-43^, Fisher exact test).

#### Intronic promoters have higher TATA box binding affinity compared to intergenic and host gene promoters

Given that intronic miRNA promoters were CpG depleted, we investigated the hypothesis that they might be mainly TATA-box promoters. To verify this we used the TRAP method [32] to calculate the overall TATA box binding affinity of each identified miRNA promoter, as described in Materials and methods. We observed that intronic promoters have a significantly higher TATA box binding affinity compared to host gene and intergenic promoters (Figure 6e, P < 10^-13 ^in both cases, Mann-Whitney U test). To understand if this condition still held when comparing intronic miRNA promoters with all human protein-coding gene promoters, we retrieved the 1,000 bp-long promoter regions upstream of all genes annotated in Ensembl. We then calculated the TATA box binding affinity for each promoter region. We found that the TATA box binding affinity for intronic promoters was still significantly higher than the affinity of all human promoters (*P *= 4.22 × 10^-17^, Mann-Whitney U test). In contrast, there was no significant difference between the binding affinity distribution of intergenic and human protein coding gene promoters (*P *= 0.68, Mann-Whitney U test).

To exclude the possibility that our results were biased by the sequence composition of intronic regions, rich in A and T nucleotides, we created two control sets: one composed of 3,000 intronic non-repetitive regions extracted randomly from the human genome, and the other represented by 1,000-bp long background sequences generated by means of a Markov model of order two, trained on the intronic promoter sequences. In both cases the TATA box affinity of intronic promoters was significantly higher than the affinity in the control regions (Figure 6f, P < 10^-15^, Mann-Whitney U test). The over-representation of TATA box binding motifs in the intronic promoter class in comparison to the host gene promoter class was also confirmed by the hit-based motif finding tool Matrix-scan [33] (*P *= 0.006, Fisher exact test). These results suggest that the number of narrow TATA-box intronic promoters is higher than expected, and that wide CpG promoters are rather exceptional inside introns of miRNA host genes.

### Intergenic, intronic and host gene miRNAs are regulated by different sets of transcription factors, suggesting different evolutionary mechanisms

Inspired by the results of the TATA box affinity analysis, we suspected that intronic promoters may have evolved differently from both intergenic and host gene promoters, therefore they would hold different regulatory elements. To verify this hypothesis and find those TFs that regulate a specific group of miRNAs, we looked at the enrichment of TFBSs from the JASPAR database [34] in the three promoter classes, by using the TRAP approach [32]. For each identified TSS we retrieved the 1 kb region centered on it, and for each set of sequences we ranked all 130 JASPAR factors according to their binding affinities for these regions. A list of significantly enriched transcription factor binding sites (TFBSs) (*P *< 0.01, Bonferroni correction) for each promoter class is given in Table S4. Our results show that host gene, intergenic and intronic promoters are indeed enriched in different sets of factors.

It has been observed that transcription factors that regulate miRNA transcription largely overlap with those that control protein coding genes (for example, p53, MYC, MYCN and REST) [3]. We found that these factors mainly regulate miRNA expression by regulating the host gene: general TFs such as REST, TP53, Myc and Mycn, in addition to SP1, Egr1, Klf4, Arnt and others (see Table S4) were enriched in host gene promoter regions, compared to the other two promoter classes. In contrast, we found that intronic promoters were enriched in TATA boxes and tissue-specific master regulator TFs such as Foxa1/Foxa2/HNF1A/HNF1B (liver factor), Pdx1 (pancreatic and duodenal homeobox), Gata1 (blood factor), Sox5 (embryonic factor), FOXF2 (lung factor), Prrx2 (skin factor) and others (see Table S4). The list of enriched factors for intergenic promoters is a combination of host gene-specific and intronic-specific factors. Half of the top TFs predicted by the TRAP tool were also confirmed by the hit-based method Matrix-scan [33] (see Additional file 1).

**Figure 7:**
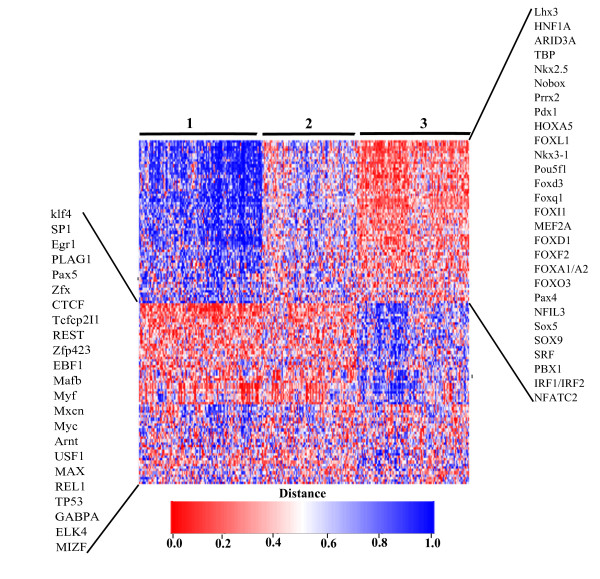
**Heatmap of transcription factor binding affinities for all identified miRNA promoters**. All identified miRNA promoters are clustered based on their affinity profiles for the 130 JASPAR core vertebrate factors. Cluster 3 is enriched in intronic promoters, while cluster 1 is enriched in host gene promoters. The transcription factors highlighted in the box on the right side are those with a high affinity for intronic promoters, which are also identified as significantly enriched by TRAP. The transcription factors in the box on the left side are those with a high affinity for host gene promoters, also reported by TRAP as significantly enriched in the same promoter class.

We clustered the identified miRNA promoters on the basis of their affinity profiles for the 130 JASPAR core vertebrate factors (Figure 7). Genes with similar affinity profiles (that is, having gained or lost affinity for the same factors) were grouped together. We found three main groups of promoters that were enriched in different TFs. Group 1 was significantly enriched in host gene promoters versus intronic and intergenic promoters (*P *< 10^-16 ^in both cases) and in intergenic promoters versus intronic promoters (*P *< 10^-16^, Figure 7). Group 2 did not show any significant enrichment. Group 3 was significantly enriched in intronic promoters (odds ratio = 2.4, *P *= 3.2 × 10^-16^, Fisher exact test) and significantly depleted in host gene and intergenic promoters (*P *= 1.1 × 10^-16 ^and *P *= 9.2 × 10^-9^, respectively). Overall, the results indicate that intergenic promoters are more similar to protein coding gene promoters, as expected. In contrast, an additional degree of freedom is provided by intronic promoters that regulate intronic miRNAs using tissue-specific transcription factors.

### Role of intronic promoters

The results presented above imply crucial differences between intronic promoters and intergenic and host gene promoters with respect to their conservation, CpG content and regulatory elements. This suggests a separate evolutionary mechanism for these promoter classes and possibly a different biological role. To further our understanding of the role of intronic promoters, especially in relation to the host gene, we studied intronic promoter usage in different tissues, the effect of intronic promoters on miRNA expression, and the relationship between intronic promoters and miRNA age.

#### Intronic promoters are expressed in a tissue-specific manner

Given the characteristics of miRNA intronic promoters, we suspected that they might be used in a tissue-specific manner. We defined a tissue-specific promoter as an identified promoter that was expressed (that is, had overlapping CAGE tags) in less than 4 out of 33 FANTOM libraries. We computed the significance of the association between intronic promoters and tissue specificity, compared to host gene promoters, by means of a Fisher exact test. This association was highly significant (odds ratio = 15.87, *P *< 2.2 × 10^-16^), suggesting that intronic promoters are used in a tissue-specific manner. Conversely, host gene promoters were significantly depleted in tissue-specific promoters and could be considered as mainly for housekeeping. We also investigated if intronic promoters were preferentially used in some tissues rather than others and found that they were significantly over-represented in brain, thymus, embryonic and lung tissues, while being significantly under-represented in T-cells, blood, monocytic cells, hepatocytes and breast and adipose tissue (Figure S4). This confirms the preferential usage of miRNA intronic promoters in certain tissues and a co-transcription of the miRNA and its host gene in others.

#### Intronic promoters potentially explain cases of poor correlation between miRNA and host gene expression

**Figure 8:**
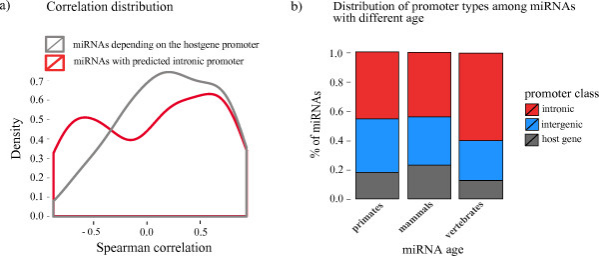
**Role of intronic promoters**. **(a) **Distribution of Spearman's correlation coefficient between the expression profiles of intragenic miRNAs and their corresponding host genes in the human brain. The gray probability density function (PDF) is for those intragenic miRNAs that are predicted to have an independent intronic promoter in the FANTOM4 brain libraries, while the red PDF is for those miRNAs that are predicted to depend on the promoter of the host gene. **(b) **Association between miRNA age and presence/absence of an independent promoter. The miRNAs are divided into three age classes according to their conservation in different lineages: vertebrates, mammals and primates. Each bar shows the fraction of intergenic, intronic and host gene promoters in each age class. Old miRNAs (conserved in vertebrates) are enriched in independent intronic promoters compared to the other two classes. miRNA, microRNA; PDF, probability density function.

Recent studies of matched mRNA-miRNA expression profiles showed instances of poor correlation between intragenic miRNAs and their corresponding host genes [10]. To investigate whether poor miRNA-host gene correlations are due to the usage of alternative intronic promoters, independent of the host gene promoter, we looked at the miRNA and mRNA expression data from the study of Somel *et al*. on 14 human brain samples at different developmental stages [35]. We divided the intragenic miRNAs in our data set into two classes: those which were associated with an intronic promoter in the FANTOM brain libraries and those that shared a promoter with the host gene. For each miRNA-host gene pair we calculated the Spearman correlation between their expression profiles, and looked at the distribution of the correlations for both classes (Figure 8a). We found that miRNAs with an independent intronic brain promoter were less positively correlated to the host gene expression, compared to host-gene dependent miRNAs (*P *= 0.08, Mann-Whitney U test). In particular, the expression of those exonic miRNAs that are predicted by our method to have an independent intronic promoter in the brain does not correlate with the host gene's expression: hsa-miR-124-1, hsa-miR-124-2, let-7a-3, let-7b, hsa-miR-433, hsa-miR-127, hsa-miR-432, hsa-miR-136 and hsa-miR-431 show very high expression levels in the brain, while their host genes are shut down, indicating that an alternative promoter may be driving their expression. This evidence implies that the condition-specific use of an intronic promoter, as an alternative to the host gene's promoter, could lead to the differential expression of an intragenic miRNA and its host gene.

#### Evolutionarily conserved miRNAs are more likely to be regulated by an intronic promoter

In addition to the effect of intronic promoters on miRNA expression level, we considered if there was a relation between the independent transcription of a miRNA and miRNA age. To investigate this, we grouped the miRNAs into three classes, young, adult and old miRNA according to their age, as described in Materials and methods. Our results, summarized in Figure 8b, indicate that there is a significant association between old miRNAs (conserved across vertebrates) and intronic promoters, compared to adult or young miRNAs (odds ratio = 1.87 and *P *= 0.0067, Fisher exact test). This suggests that intragenic old miRNAs, unlike adult and young miRNAs, are more likely to be regulated by their own independent promoter, rather than depending on the host gene promoter (Figure 8b), suggesting the divergent evolution of intragenic miRNAs and their host genes. In addition, old miRNAs seem to be enriched in non-coding transcripts compared to protein-coding transcripts (fold enrichment 4.5, *P *= 4.3 × 10^-8^, Fisher exact test). This evidence might be related to the host gene's function and to possible feedback loops between the miRNA and the host. However, a much more detailed analysis of host gene function and miRNA targets and host-target interactions are needed to support this hypothesis.

## Discussion

Identifying the exact location of miRNA promoters is the critical first step towards annotating full miRNA primary transcripts, predicting the transcription factors that regulate them and, ultimately, explaining miRNA function in complex regulatory networks. Our study has two main contributions: we introduce a new strategy to annotate miRNA promoters using high-throughput genomic data and we apply it to study the characteristics and activity of intronic miRNA promoters.

Our model successfully integrates deepCAGE data with other promoter features to score the potential of candidate regions for being real miRNA TSSs. Despite the transient nature of miRNA primary transcripts, we show that deepCAGE data from several tissues can be used systematically to identify miRNA promoters with high coverage by exploiting the fact that most miRNA primary transcripts are capped, and that miRNA expression patterns, as well as degradation rates, are tissue and condition specific. The high coverage of the method is demonstrated by the fact that we can assign a putative promoter to 82% of annotated miRNAs.

A miRNA promoter classification method using high-throughput sequencing data was recently proposed by Chien *et al. *[16]. However, their model is a supervised approach trained on protein-coding gene features and is based on the assumption that miRNA and protein-coding gene promoters have identical characteristics. Although previous studies have shown that the transcription of miRNA genes is regulated in a similar manner to that of protein coding genes [3], mRNAs and pri-miRNAs follow different post-transcriptional pathways, have different lifetimes and, most likely, different read count distributions in the TSS region. Assuming that read counts, which are a measure of expression at the promoter level, are similar for protein-coding genes and miRNAs might lead to a high number of false positives. In addition, given the small number of validated pri-miRNA TSSs, we claim that a semi-supervised model, which does not include protein-coding gene features in the learning process, is more appropriate for describing miRNA promoters.

Our method is able to verify known miRNA TSSs and the remaining identified TSSs are strongly supported by PolII ChIP-seq data, as well as full-length primary transcripts from RNA-seq data. In addition, it compares well with existing chromatin-based methods, confirming most of the annotations reported previously. One potential limitation of our method is that it might not be sensitive enough to detect transient pri-miRNAs whenever the Drosha-processing step happens very quickly and few or no reads can be associated with a pri-miRNA TSS. This limitation could be overcome by chromatin-based methods, which have been successfully used over the past few years to detect active promoters [5]. However, compared to histone modification-based methods, deepCAGE data offers the possibility of identifying all possible alternative promoters, producing a direct readout of promoter activity, and thus aiding in detecting intronic miRNA promoters that are preferentially used in a tissue-specific manner. Compared to previous studies, we found an increased number of miRNAs that are predicted to be regulated by their own independent intronic promoter. This number increased from 30% [9,10] to 50%, if we consider only those promoters that could be validated by PolII and/or RNA-seq coverage.

Previous studies indicate that some unknown proportion of CAGE tags falling within transcripts are re-capped products of spliced long RNAs [36]. Although most of these unusual tags map to internal exons or cross exon-exon junctions, and are therefore excluded by our method, some of them might fall within intergenic or intronic regions upstream of annotated miRNAs. Such re-capping products, if any, can give rise to wrongly classified promoters and might represent one of the reasons for the observed lower precision of PROmiRNA in detecting intergenic and intronic miRNAs compared to host gene promoters.

In addition, it is important to stress that it is not yet fully understood how miRNA biogenesis and the splicing process of intragenic transcripts interact with each other. Due to the nature of the data used here, our method can be used to identify the start sites of putative primary transcripts but it does not provide many clues for how and when splicing of primary transcripts or miRNA-containing introns happens. Cross-talk between the splicesome and the Microprocessor complex has been hypothesized in previous studies [22,23,37] and here we show that exonic miRNAs, as well as mirtrons, can be transcribed by an independent promoter, suggesting that the relationship between miRNA processing and splicing might be more complex than expected. The splicing-dependent processing of exonic miRNAs, as well as mirtrons, is an interesting possibility but too speculative to be discussed here.

While this manuscript was in preparation, new CAGE data from the RIKEN Institute were released by the ENCODE project. We therefore checked our miRNA promoters against the ENCODE-generated CAGE TSSs. The details of this comparison are presented and discussed in Additional file 1. Overall the results indicate that, although almost 100% of our identified miRNA host gene promoters are also annotated in ENCODE, the ability of their model to recover our independent miRNA promoters drops to about 60% and 50% for intergenic and intronic promoters, respectively.

Beyond the development of the method, we are also able to identify several unique characteristics of intronic promoters. We performed a large-scale analysis of miRNA promoter features for both intergenic and intragenic miRNAs, and we can identify the unique characteristics that distinguish intronic promoters from host gene and intergenic promoters. This allows us to give a partial explanation of the role of intronic promoters, as well as to relate them to the evolution of different miRNA classes. Our results show that CpG islands are primarily a feature of wider host gene promoters, while intergenic and especially intronic promoters are mainly low CpG promoters. Additionally, intronic promoters are usually narrow (less than 100 bp) and enriched in TATA box elements. Our analysis also shows that intronic promoters tend to be regulated by a different set of transcription factors, compared to intergenic and host gene promoters, and that these factors are enriched in tissue-specific master regulators. This has two implications. First, the genomic features of intronic promoters, that is low CpG content and TFBSs for tissue-specific master regulators, suggest that they are alternative promoters that modulate miRNA expression in a tissue or cellular state-specific manner. This is further confirmed by our analysis of miRNA promoter usage among all the FANTOM4 libraries: intronic promoters are mainly tissue-specific, unlike their host genes, and are used in an alternative manner in complex tissues such as the brain. The second implication is that the differences in regulatory elements between intragenic and host gene promoters point towards a different evolution of the two promoter classes, although additional investigation is needed to fully understand these evolutionary differences.

The strong bias for miRNAs to be oriented on the same strand as their host gene might suggest that they are co-regulated. However, previous investigations indicated that several intragenic miRNAs have inconsistent expression patterns with their host genes, and that only a minority of them are co-expressed with the host gene [10]. Here we have observed several instances of poor or negative correlation of matched miRNA and mRNA expression in brain tissue. Although differences in host gene and mature miRNA expression could be due to differential miRNA processing or post-transcriptional regulation [11,38], we tested the hypothesis that intronic promoters are in part responsible for the host gene-independent expression of miRNAs and they are not just transcriptional noise. We could indeed show that miRNAs with intronic promoters are significantly less positively correlated to their host genes, and that instances of poor correlation can be attributed to the use of intronic promoters. The notion of intronic promoters driving expression of intragenic miRNAs has been hypothesized before, but this is the first time, to our knowledge, that a statistically significant association between expression patterns and intronic promoters has been demonstrated.

Finally we show that old miRNAs are unlikely to be co-transcribed with their host gene, and the majority of them have developed an independent intronic promoter. It has been hypothesized that introns are 'hot spots' for the emergence of novel miRNAs, as they are particularly suitable for evolving hairpin structures from already transcribed RNAs. Consistent with this hypothesis, younger miRNAs are more often located in introns than ancient ones [39]. In spite of that fact, we also observe that more than half of the old miRNAs are located inside introns and are significantly depleted in GC content compared to young miRNAs (*P *= 1.092 × 10^-8^, Wilcoxon test, two sided), in agreement with previous studies [40]. We speculate that old miRNAs might be functionally important, and therefore modulate the activity of target genes that are crucial for certain biological processes. Our observation fits the evolutionary model proposed by Chen and Rajewsky [41], according to which novel non-conserved miRNAs are initially expressed at low level and in specific tissues, to limit the deleterious effect that they might have on their targets. We hypothesize that once such miRNAs complete what they define as the purging phase of evolution and become old, they acquire a defined and important functional role in regulating some specific targets, therefore developing their own promoter. This would ensure that they are able to perform their function independently of the host gene, which protects them from mutations that might occur in the promoter of the host gene and allows them to remain functional in situations where the host gene is shut down.

## Conclusions

The systematic discovery and analysis of miRNA promoters presented in this study is another step towards understanding miRNA biogenesis, in particular the transcriptional control of intragenic miRNAs, as well as the way in which a novel miRNA promoter can emerge and evolve to acquire a specialized function.

## Materials and methods

### Data sets

#### Human miRNAs and gene annotation

All annotated human miRNAs and their genomic coordinates were obtained from miRBase release 18. *Homo sapiens *gene annotation (GRCh37) was obtained from Ensembl v66. We separated the annotated miRNAs into two groups: intergenic (if they were contained in intergenic regions between annotated genes) or intragenic (if the miRNA overlapped with an Ensembl gene, referred to in this case as an miRNA host gene).

#### DeepCAGE data from FANTOM4

CAGE is used to profile gene TSSs genome-wide by mapping tags from the 5' end of RNA transcripts to the genome. These tags are extracted by a combination of cap trapping and cleavage by restriction enzymes [42]. CAGE was previously applied to different RNA libraries in human and mouse, allowing the study of general promoter properties and the analysis of promoter usage in different tissues [21]. An extension of CAGE, called deepCAGE, combines CAGE technology with deep sequencing [43]. Within the FANTOM4 project this method was used to define promoters in mouse as well as human across several tissues and conditions [17,44]. We downloaded pre-mapped CAGE tags from 33 human RNA libraries from FANTOM4, corresponding to several tissues, conditions and developmental stages. Libraries corresponding to technical replicates were excluded from the analysis. As the reads provided on the FANTOM4 website [17] were mapped on the hg18 human assembly, we re-mapped the read coordinates to the hg19 assembly using the LiftOver tool from UCSC. The number of mapped reads in each library ranged from 17,000 up to 2 million, resulting in a total tag count of approximately 22 million CAGE tags (Table S1 in Additional file 1). Only CAGE tags mapping to unique positions in the genome were considered for further analysis.

#### PolII ChIP-seq data

Bed files corresponding to already processed PolII peaks from several cell lines were downloaded from the HAIB track inside the ENCODE project (see the details in Table S2 in Additional file 1).

#### Human tissue RNA-seq data

RNA-seq data consisting of 100 bp long reads from 16 different RNA libraries, which were generated as part of the Human Body Map 2.0 Project, were downloaded from GEO (GEO ID: GSE30611). The data was generated from three different types of libraries (polyA-selected, poly-A selected with normalization and no-polyA mRNA) and from two different flow cells, named FCA and FCB. Each library is a mixture of 16 different human tissues (Table S3 in Additional file 1).

#### miRNA and mRNA brain expression data

miRNA expression levels, measured using the small RNA-seq technique, were taken from Somel *et al. *[35]. Data are available for ten human brain samples taken during development and aging. The mRNA expression levels of the corresponding host genes (for the same human brain samples) were taken from Liu *et al. *[45] and were measured using RNA-seq.

### Data normalization

For each of the 33 FANTOM4 libraries used in this study, and for each annotated pre-miRNA, we selected those genomic positions up to 50 kb upstream of the miRNA where at least one read was mapped. For each genomic position we computed a tag count vector whose elements are the read counts in that position for each RNA library. As the libraries strongly differ in terms of the total number of mapped reads, to take into account systematic variations between samples that might cause the absolute top counts to vary across the experiments, we applied per-position quantile normalization, inspired by the quantile normalization procedures successfully applied to micro-array data. Position-specific tag counts from each sample, which can be interpreted as expression values at the promoter level at 1 bp resolution, were transformed to match a common reference distribution randomly chosen between the 33 libraries. Quantile normalization has already been used and shown to perform quite well when applied to RNA-seq data for differential expression quantification [46]. Furthermore, a similar procedure has been applied to normalize deepCAGE data, where the reverse-cumulative reference distribution for tag counts is explicitly modeled by a power law [44].

### Core promoter construction and filtering

For each individual library, we grouped the tags into clusters if the overlap between their mapped genomic coordinates was at least 1 bp. These clusters were used to define putative transcription start regions. We then summed the normalized tag counts inside each cluster. We defined intergenic promoters as those tag clusters that were located in intergenic regions upstream of annotated intergenic miRNAs. Host gene promoters were defined as those tag clusters whose genomic positions overlapped with the start sites of annotated transcripts of intragenic miRNA host genes. Intronic promoters were defined as those tag clusters located in the same intron, or a different intron, of an intragenic miRNA host gene. Tag clusters whose genomic positions overlapped with the TSSs of other annotated Ensembl transcripts were filtered out, because they did not correspond to miRNA TSSs. Tags spanning annotated exons of the same genes were also excluded from further analysis, as most probably they correspond to alternative transcripts of the host gene.

### The model for promoter classification

Our probabilistic framework is based on a partially supervised mixture model for classifying candidate regions enriched in CAGE tags as either putative promoters or background noise. Given that there is no benchmark data set of validated miRNA promoters, and very few pri-miRNA transcripts are annotated in genomic databases, the use of a supervised learning method did not seem feasible. Therefore, we opted for a semi-supervised method where the probabilistic distribution of the data is represented by a mixture model of putative promoters versus background regions, and where the optimal separation between the two classes was learned directly from the data. Specifically, our mixture model uses tag counts and computed promoter features to cluster the data into two classes, promoters and non-promoters, and calculate the posterior probability that a certain region belongs to one or the other class.

The use of a mixture model was motivated by two main aspects: the distribution of the log of CAGE tag counts, from all pooled RNA libraries considered in this study, shows an approximately bimodal distribution (Figure S3a in Additional file 1). Second, the use of a mixture model avoided using *ad hoc *thresholds to define regions that are sufficiently enriched in CAGE tags to be considered as real transcription start sites.

Mixture modeling associates each cluster with a model component, which is defined by the underlying distribution of the estimated data. In detail, if **X **represents the observed data generated by continuous random variables *X*_1_...*X_N,_*, **Z **a set of latent variables having a 1-of-K representation, and *θ *a vector of unknown parameters, along with a likelihood function *p*(**X**, **Z**|*θ*), the distribution over the observed variables can be written as a linear combination of probability distributions over the *K *classes [47]. In our case **X **represents the mixture of putative promoters and background regions from all pooled RNA libraries and *K *= 2, as we aim at producing a binary classification of promoter/non-promoter. There are therefore only two possible states for **Z**, **Z**_1 _= 1 for promoters and **Z**_2 _= 1 for background regions.

(1) $$p\left(X|\theta \right)=\underset{Z}{\sum }p\left(X,Z|\theta \right)=\overset{N}{\underset{i=1}{\prod }}\left(\underset{k}{\sum }\pi_{k}\cdot p_{k}\left(X_{i}|\theta_{k}\right)\right)$$

The π_k _are called mixing proportions or priors and are such that the marginal distribution over **Z **is specified in terms of the mixing coefficients:

(2) $$p\left(Z_{k}=1\right)=\pi_{k}$$

More specifically, the total number of CAGE tags in a certain region *i*, *x_i_*, is modeled with an inverse Gaussian distribution, using different sets of parameters for the promoter and the background class:

(3) $$p\left(X_{i}|Z_{k}\right)=\text{Inverse Gaussian}\left(X_{i}|\mu_{k},\lambda_{k}\right)=\left(\frac{\lambda_{k}}{2\pi X_{i}^{3}}\right)\cdot \text{exp}\left(\frac{-\lambda_{k}{\left(X_{i}-\mu_{k}\right)}^{2}}{2\mu_{k}^{2}X_{i}}\right)$$

where *k *= 1 for the promoter class and *k *= 2 for the background class. While a Poisson distribution or a negative binomial distribution is usually the choice to model read count data, the inverse Gaussian distribution allowed us to model continuous values more accurately, such as quantile-normalized tag counts, and especially to take into account long tails in the tag count distribution due to the high numbers of reads mapping to highly expressed promoter regions.

In unsupervised mixture modeling, the input is only the data **X **and the cluster labels are unknown, that is we do not know in advance if a certain region belongs to the promoter or the background class. In this case the expectation maximization (EM) algorithm is used for parameter estimation [47]. While it is reasonable to assume that the high read count mode in Figure 3Sa is enriched in true miRNA promoters, the low read count mode might contain noise as well as lowly expressed promoters, which risk being wrongly classified as noise if no further information is used in the model to discriminate between the two classes. Previous studies observed high levels of stochastic background transcription in CAGE data, corresponding to few tags mapping to a specific region and representing noise without biological significance [44]. If this is true for highly expressed protein-coding genes, for a miRNA TSS region one or few mapped tags may correspond to a real TSS, and the low tag count could be due to fast pri-miRNA degradation, rather than low expression. Therefore, we introduced some prior knowledge into the model in the form of a prior probability. The prior probability is based on several significant sequence features, and allowed a better discrimination between real TSSs with a low number of associated tag counts and noise.

Formally, we assumed that some knowledge is available for a subset of observations, and, inspired by the belief-based mixture model in Szczurek *et al. *[48], for each candidate region we regarded the sequence features as our belief that that region is a real promoter. We set an equivalent of the prior *π_ik _*differently for each example *X_i_*, where *i *= 1...*N*, *N *being the number of observations, to handle imprecise knowledge about the examples. The belief itself is a probability distribution over the promoter and background classes given by a vector *π_i_*, satisfying

(4) $$\underset{k}{\sum }\pi_{ik}=1$$

We modeled the prior probability of a certain region *i *of being a promoter *p*(prom) = *π*_*i*1 _as a logistic function of several sequence features, in a similar way to Pique-Regi *et al*. in the software CENTIPEDE [49]:

(5) $$\pi_{i1}=\frac{1}{1+{\text{e}}^{-y_{i}}}$$

(6) $$y_{i}=\beta_{0}+\beta_{1}\cdot \text{Cp}{\text{G}}_{i}+\beta_{2}\cdot \text{con}{\text{s}}_{i}+\beta_{3}\cdot \text{TAT}{\text{A}}_{i}+\beta_{4}\cdot \text{mirna proximit}{\text{y}}_{i}$$

where CpG*_i _*is the normalized CpG content in the genomic region *i *of length *L *around the candidate TSS, computed as the ratio of observed over expected CG dinucleotides:

(7) $$\text{Cp}{\text{G}}_{i}=\frac{CG/L}{{\left(\left(C+G\right)/2L\right)}^{2}}$$

cons*_i _*is the average PhastCons conservation score of region *i *computed from the alignment of 46 vertebrate genomes taken from the UCSC Genome Browser; TATA*_i _*is the affinity score of region *i *for a TATA box element computed with the TRAP tool, and mirna proximity*_i _*measures the proximity of the candidate TSS to the mature miRNA. The prior probability of being a background region is then *p*(bg) = 1 - *π*_*i*1 _= *π*_*i*2_.

The parameters of the model (*μ*_1_, *λ*_1_, *μ*_0 _and λ_0_) were determined by maximizing the likelihood function in (1) using the EM algorithm. The *β *parameters from the prior probability were set in advance and determined by means of a logistic regression model on a few available miRNA promoter and background region examples (see Additional file 1 for a detailed description). Once the model had converged, the final posterior probability (or conditional probability of **Z **given the data **X**) of belonging to the promoter/background class, given the evidence, could be found using Bayes' theorem:

(8) $$p\left(Z_{ik}|X_{i}\right)=\frac{p\left(X_{i}|Z_{ik}=1\right)\cdot \pi_{ik}}{\underset{k}{\sum }p\left(X_{i}|Z_{ik}=1\right)\cdot \pi_{ik}}$$

A new candidate region *x*_0 _can be easily tested by computing

(9) $$p\left(\text{prom}|x_{0}\right)=\frac{\pi_{1}\left(x_{0}\right)p\left(x_{0}|Z_{01}=1\right)}{\underset{k}{\sum }\pi_{k}\left(x_{0}\right)p\left(x_{0}|Z_{0k}=1\right)}$$

#### Construction of the training set

To help the algorithm in the learning process, we constructed a set of background sequences by extracting them randomly from intergenic non-repetitive regions in the human genome. For such sequences we also determined CAGE tag counts and sequence properties as described above. This set of observations was used as exact examples (the negative set) for our algorithm: their cluster labels were known and remained fixed during the parameter estimation with the EM algorithm, that is the conditional probabilities for such observations were set to *p*(bg) = 1.0 and 0.0 *p*(prom) = 0.0.

#### Filtering of the identified promoters

As the same identified promoter can occur in more than one tissue or regulate more than one miRNA, when miRNAs occur in genomic clusters, a non-redundant set of representative promoter sequences was compiled using the cd-hit software [50]. Specifically, sequences were clustered with a similarity threshold of 0.8 and the promoter with the highest count of mapped tags was chosen as representative for each cluster.

### Estimation of the method performance based on PolII ENCODE ChIP-seq data

All processed peaks from the ChIP-seq libraries in the HAIB ENCODE track were pooled together (see Table S2 in Additional file 1). The 1,000 bp-long regions surrounding candidate miRNA TSSs overlapped with PolII peak regions. If the cutoff for the promoter posterior probability is *c*, the numbers of true positives (TP), true negatives (TN), false positives (FP) and false negatives (FN) were determined in the following way: an identified miRNA promoter (*p*(prom) >*c*) overlapping a PolII peak region was considered to be a true positive; an identified miRNA promoter not overlapping a PolII peak was considered to be a false positive; a region not classified as a promoter (*p*(prom) <*c*) was considered to be a true negative if it did not overlap a PolII peak and as false negative if it overlapped a PolII peak. A receiver operating curve (ROC) was built by varying the cutoff *c *for *p*(prom) and the precision was determined at recalls of 71%, 60%, 22% and 21%, corresponding to *p*(prom) = *c *= 0.5 >*p*(bg), for all, host gene, intergenic and intronic promoter classes, respectively.

### Estimation of the method precision based on RNA-seq coverage

We extracted genomic regions starting at the identified miRNA TSS and ending 70 bp after the annotated mature miRNA. These regions corresponded, approximately, to full-length miRNA transcripts. For each region we computed the coverage of RNA-seq reads using the 16 Human Body Map strand-specific read libraries. We defined the pri-miRNA coverage as the fraction of bases continuously covered by mapped reads, computed using the coverageBed program in Bedtools [51]. Each identified region was associated with 16 coverage values, one for each RNA-seq library. To decide if a certain pri-miRNA transcript was present in the RNA-seq data, we tested intergenic and intronic pri-miRNAs separately and compared their read coverage values to a background distribution. For intergenic primary transcripts the background distribution of the read coverage was computed from 10,000 randomly selected intergenic regions, while for intragenic primary transcripts the background was built from 10,000 random intronic regions. A Kolmogorov-Smirnov test was used to assess the significance of the read coverage enrichment of a putative pri-miRNA compared to the background. An identified primary transcript was defined as present, that is considered to be a true positive, if it had a coverage fold enrichment of at least 2.0 compared to the average background and an adjusted *P *value from the Kolmogorov-Smirnov test lower than 0.1. A primary transcript was defined as absent, that is false positive, if it did not satisfy the criteria above. The precision of the method was then defined as prec = TP / (TP + FP). An estimation of the number of false negatives from the RNA-seq data would require the identification of all possible transcripts from all libraries, included the pri-miRNA transcripts that are missed from our method. The complete transcriptome analysis of all the RNA-seq libraries from the Human Body Map 2.0 Project is beyond the scope of this work. For this reason we limited the RNA-seq-based evaluation of PROmiRNA to the estimation of true positives and false positives based on the read coverage enrichment in the identified pri-miRNA regions.

### Transcription factor binding site (TFBS) analysis

#### TF binding site affinity enrichment with TRAP

We calculated the enrichment of a transcription factor for a set of 1 kb long promoter sequences using the TRAP method. TRAP (Transcription Factor Affinity Prediction) uses a physical model to predict the relative binding affinities of TFs to DNA regulatory regions [52]. To compare the affinities of different transcription factors for the same sequence, normalized affinities and associated *P *values were calculated as described in [53]. In this analysis we used all 130 core vertebrate TFs from the JASPAR database, and the analysis was performed independently for intergenic, intronic and host gene promoters. We ran the TRAP program with default parameters, as described in [32], producing a list of transcription factors ranked according to their normalized affinity for the set of sequences under study. The enrichment in binding affinity was computed, in all cases, with respect to a background set of protein-coding gene promoter sequences.

#### Hit-based TF sequence scanning

We used the pattern-matching program Matrix-scan from the RSAT suite [33] to scan the identified miRNA promoter sequences and search for hits of TFBSs, based on position-specific scoring matrices, in the 1 kb long regions surrounding the identified TSS using the same JASPAR factors.

### Determination of miRNA age

To determine human miRNA ages, we searched for orthologs of all annotated mature human miRNAs in the genomes of 11 species: chimpanzee, gorilla, orangutan, rhesus macaque, marmoset, mouse, rat, dog, cow, opossum and chicken. The genome sequences for these organisms were downloaded from UCSC. Specifically, we mapped miRNA precursors to each genome using reciprocal BLAST or reciprocal LiftOver with default settings, and required the length of the hit sequence to be more than 60% and less than 130% of the query sequence. We then extracted mature miRNA orthologs from the precursor sequence alignment made using the Muscle sequence alignment algorithm [54]. Next, we classified miRNAs into three categories based on their orthologous status: the first class included human miRNAs with orthologs only in primate species, referred to as young miRNAs; the second class included miRNAs with at least one ortholog in primate species and at least one ortholog in mammal species, referred to as adult miRNAs and the third class included miRNAs that had at least one ortholog in the vertebrate species, referred to as old miRNAs.

### Plasmid constructs and luciferase assays

Fragments of approximately 500 bp upstream of the putative transcriptional start site were cloned into pGL3-basic 5-prime to the Luciferase gene. HEK293 cells were seeded in 96-well plates and co-transfected with 0.1 μg of pGL3-basic-miRNA promoter construct and 0.02 μg of pRL-TK vector (Promega) expressing the *Renilla reniformis *Luciferase as a transfection control. All transfections were carried out in triplicate, and all experiments were done at least three times. Luciferase activity was measured 24 h post transfection using the Dual-Luciferase Reporter Assay System kit (Promega) according to the manufacturer's protocol.

## List of abbreviations used

bp: base pair; CAGE: cap analysis of gene expression; EM: expectation maximization; EST: expressed sequence tag; kb: kilobase; miRNA: microRNA; PDF: probability density function; pre-miRNA: precursor miRNA; pri-miRNA: primary miRNA; ROC: receiver operating curve; TF: transcription factor; TFBS: transcription factor binding site; TSS: transcription start site.

## Competing interests

The authors declare that they have no competing interests.

## Authors' contributions

AM conceived the study, collected the data, implemented the method, performed the analysis and wrote the manuscript. MH and HH provided additional data, helped in the analysis and with the interpretation of the results. JL helped to conceive and implement the statistical model. DV and AM performed the experiments. UAO provided valuable biological expertise and drafted the experimental validation section of the manuscript. MV supervised the study. MH, JL, UAO and MV commented on the manuscript at all stages. All authors have read and approved the manuscript for publication.

## Supplementary Material

Additional file 1 **This file contains a detailed description of the data set used, the method and additional results**. Click here for file

Additional file 2 **This file contains a list of all identified human miRNA promoters, together with their genomic coordinates, computed posterior probabilities, regulatory features, tissue of expression and miRNA age**. Click here for file

Additional file 3 Click here for file

## References

[B1] Schanen BC Li X Transcriptional regulation of mammalian miRNA genes Genomics 2011 14 1 6 10.1016/j.ygeno.2010.10.005 20977933 PMC3019299

[B2] Davis BN Hata A Regulation of MicroRNA Biogenesis: A miRiad of mechanisms Cell Commun Signal 2009 14 7 18 10.1186/1478-811X-7-18 PMC3224893 19664273

[B3] Krol J Loedige I Filipowicz W The widespread regulation of microRNA biogenesis, function and decay Nat Rev Genet 2010 14 597 610 20661255 10.1038/nrg2843

[B4] Fickett J Hatzigeorgiou A Eukaryotic Promoter Recognition Genome Res 1997 14 861 878 9314492 10.1101/gr.7.9.861

[B5] Zeng J Zhu S Yan H Towards accurate human promoter recognition: a review of currently used sequence features and classification methods Brief Bioinform 2009 14 498 508 10.1093/bib/bbp027 19531545

[B6] Sonnenburg S Zien A Raetsch G ARTS: accurate recognition of transcription starts in human Bioinformatics 2006 14 e472 480 10.1093/bioinformatics/btl250 16873509

[B7] Wang X Zhao X Li Y Zhang M High-resolution human core-promoter prediction with CoreBoost HM Genome Res 2009 14 266 275 18997002 10.1101/gr.081638.108 PMC2652208

[B8] Megraw M Pereira F Jensen S Ohler U Hatzigeorgiou A A transcription factor affinity-based code for mammalian transcription initiation Genome Res 2009 14 644 656 10.1101/gr.085449.108 19141595 PMC2665783

[B9] Ozsolak F Poling LL Wang Z Liu H Liu XS Roeder RG Zhang X Song JS Fisher DE Chromatin structure analyses identify miRNA promoters Gene Dev 2008 14 3172 3183 10.1101/gad.1706508 19056895 PMC2593607

[B10] Monteys AM Spengler RM Wan J Tecedor L Ka KAL Xing Y Davidson BL Structure and activity of putative intronic miRNA promoters RNA 2010 14 495 505 10.1261/rna.1731910 20075166 PMC2822915

[B11] Ladewig E Okamura K As AF Westholm J Lai E Discovery of hundreds of mirtrons in mouse and human small RNA data Genome Res 2012 14 1634 1645 10.1101/gr.133553.111 22955976 PMC3431481

[B12] Barski A Jothi R Cuddapah S Cui K Roh T Schones DE Zhao K Chromatin poises miRNA- and protein-coding genes for expression Genome Res 2009 14 1742 1751 10.1101/gr.090951.109 19713549 PMC2765269

[B13] Marson A Levine SS Cole MF Frampton GM Brambrink T Johnstone S Guenther MG Johnston WK Wernig M Newman J Calabrese JM Dennis LM Volkert TL Gupta S Love J Hannett N Sharp PA Bartel DP Jaenisch R Young RA Connecting microRNA genes to the core transcriptional regulatory circuitry of embryonic stem cells Cell 2008 14 521 533 10.1016/j.cell.2008.07.020 18692474 PMC2586071

[B14] Saini HK Griffiths-Jones S Enright AJ Genomic analysis of human microRNA transcripts Nat Acad Sci Proc 2007 14 17719 17724 10.1073/pnas.0703890104 PMC2077053 17965236

[B15] Zhou X Ruan J Wang G Zhang W Characterization and Identification of microRNA core promoters in four model species Plos Comput Biol 2007 14 e37 10.1371/journal.pcbi.0030037 17352530 PMC1817659

[B16] Chien CH Sun YM Chang WC Anf TY Lee PYCH Tsai WC Horng JT Tsou AP Huang HD Identifying transcriptional start sites of human microRNAs based on high-throughput sequencing data Nucleic Acid Res 2011 14 9345 9356 10.1093/nar/gkr604 21821656 PMC3241639

[B17] Kawaji H Severin J Lizio M Waterhouse A Katayama S Irvine KM Hume DA Forrest ARR Suzuki H Carninci P Hayashizaki Y Daub CO The FANTOM web resource: from mammalian transcriptional landscape to its dynamic regulation Genome Biol 2009 14 R40 10.1186/gb-2009-10-4-r40 19374775 PMC2688931

[B18] Kozomara A Griffiths-Jones S miRBase: integrating microRNA annotation and deep-sequencing data Nucleic Acids Res 2011 14 D152 D157 10.1093/nar/gkq1027 21037258 PMC3013655

[B19] Relle M Becker M Meyer R Stassen M Schwarting A Intronic promoters and their noncoding transcripts: A new source of cancer-associated genes Mol Carcinog 2012 doi: 10.1002(mc.21955) 10.1002/mc.21955 22930395

[B20] PROmiRNA http://promirna.molgen.mpg.de

[B21] Carnici P Sandelin A Lenhard B Katayama S Shimokawa K Ponjavic J Semple CAM Taylor MS Engstrom PG Frith MC Forrest ARR Alkema WB Tan SL Plessy C Kodzius R Ravasi T Kasukawa T Fukuda S Kanamori-Katayama M Kitazume Y Kawaji H Kai C Nakamura M Konno H Nakano K Mottagui-Tabar S Arner P Chesi A Gustincich S Persichetti F Genome-wide analysis of mammalian promoter architecture and evolution Nat Genet 2006 14 626 635 10.1038/ng1789 16645617

[B22] Kim V Han J Siomi M The widespread regulation of microRNA biogenesis, function and decay Nat Rev Mol Cell Biol 2009 14 126 39 10.1038/nrm2632 19165215

[B23] Kim Y Kim V Processing of intronic microRNAs EMBO J 2007 14 775 783 10.1038/sj.emboj.7601512 17255951 PMC1794378

[B24] Taganov K Boldin M Chang K Baltimore D NF-κB-dependent induction of microRNA miR-146, an inhibitor targeted to signaling proteins of innate immune responses Proc Natl Acad Sci 2006 14 12481 12486 10.1073/pnas.0605298103 16885212 PMC1567904

[B25] Eis P Tam W Sun L Chadburn A Gomez M Lund E Dahlberg J Accumulation of miR-155 and BIC RNA in human B cell lymphomas Proc Natl Acad Sci 2003 14 3627 3632 10.1073/pnas.0500613102 PMC552785 15738415

[B26] Chang TC Wentzel EA Kent OA Ramachandran K Mullendore M Lee KH Feldmann G Yamakuchi M Ferlito M Lowenstein CJ Arking DE Beer MA Maitra1 A Mendell JT Transactivation of miR-34a by p53 broadly influences gene expression and promotes apoptosis Mol Cell 2007 14 745 752 10.1016/j.molcel.2007.05.010 17540599 PMC1939978

[B27] Cai X Hagedorn C Cullen B Human microRNAs are processed from capped, polyadenylated transcripts that can also function as mRNAs RNA 2004 14 1957 1966 15525708 10.1261/rna.7135204 PMC1370684

[B28] Saito Y Liang G Egger G Friedman J Chuang J Ga GC Jones P Specific activation of microRNA-127 with downregulation of the proto-oncogene BCL6 by chromatin-modifying drugs in human cancer cells Cancer Cell 2006 14 435 443 10.1016/j.ccr.2006.04.020 16766263

[B29] Wu TD Nacu S Detection of redundant fusion transcripts as biomarkers or disease-specific therapeutic targets in breast cancer Cancer Res 2012 14 1921 1928 10.1158/0008-5472.CAN-11-3142 22496456

[B30] Saxonov S Berg P Brutlag D A genome-wide analysis of CpG dinucleotides in the human genome distinguishes two distinct classes of promoters Proc Natl Acad Sci 2006 14 1412 1417 10.1073/pnas.0510310103 16432200 PMC1345710

[B31] Fujita S Iba H Putative promoter regions of miRNA genes involved in evolutionary conserved regulatory systems among vertebrates Bioinformatics 2007 14 303 308 18055479 10.1093/bioinformatics/btm589

[B32] Thomas-Chollier M Hufton A Heinig M O'Keeffe S Masri N Roider H Manke T Vingron M Transcription factor binding predictions using TRAP for the analysis of ChIP-seq data and regulatory SNPs Nat Protoc 2011 14 1860 1869 10.1038/nprot.2011.409 22051799

[B33] Turatsinze JV Thomas-Chollier M Defrance M van Helden J Using RSAT to scan genome sequences for transcription factor binding sites and cis-regulatory modules Nat Protoc 2008 14 1578 1588 10.1038/nprot.2008.97 18802439

[B34] Portales-Casamar E Thongjuea S Kwon AT Arenillas D Zhao X Valen E Yusuf D Lenhard B Wasserman WW Sandelin A JASPAR 2010: the greatly expanded open-access database of transcription factor binding profiles Nucleic Acids Res 2010 14 D105 D110 10.1093/nar/gkp950 19906716 PMC2808906

[B35] Somel M Guo S Fu N Hu HY Xu Y Yuan Y Ning Z Hu Y Menzel C Hu H Lachmann M Zeng R Chen W Khaitovich P MicroRNA, mRNA, and protein expression link development and aging in human and macaque Genome Res 2010 14 1207 1218 10.1101/gr.106849.110 20647238 PMC2928499

[B36] Project ASHET Post-transcriptional processing generates a diversity of 5´-modified long and short RNAs Nature 2009 14 1028 1032 10.1038/nature07759 19169241 PMC2719882

[B37] Kataoka N Fujita M Ohno M Functional association of the Microprocessor complex with the spliceosome Mol Cell Biol 2009 14 3243 3254 10.1128/MCB.00360-09 19349299 PMC2698730

[B38] Winter J Jung S Keller S Gregory RI Diederichs S Many roads to maturity: microRNA biogenesis pathways and their regulation Nat Cell Biol 2009 14 228 234 10.1038/ncb0309-228 19255566

[B39] Berezikov E Evolution of microRNA diversity and regulation in animals Nat Rev Genet 2011 14 846 860 10.1038/nrg3079 22094948

[B40] Sun J Zhou M Mao Z Li C Characterization and evolution of microRNA genes derived from repetitive elements and duplication events in plants PLoS One 2012 14 e34092 10.1371/journal.pone.0034092 22523544 PMC3327684

[B41] Chen K Rajewsky N The evolution of gene regulation by transcription factors and microRNAs Nat Rev Genet 2007 14 93 103 17230196 10.1038/nrg1990

[B42] Shiraki T Kondo S Katayama S Waki K Kasukawa T Kawaji H Kodzius R Watahiki A Nakamura M Arakawa T Fukuda S Sasaki D Podhajska A Harbers M Kawai J Carninci P Hayashizaki Y Cap analysis gene expression for high-throughput analysis of transcriptional starting point and identification of promoter usage Nat Acad Sci Proc 2003 14 15776 15781 10.1073/pnas.2136655100 PMC307644 14663149

[B43] de Hoon M Hayashizaki Y Deep cap analysis gene expression (CAGE): genome-wide identification of promoters, quantification of their expression, and network inference Biotechniques 2008 14 627 632 18474037 10.2144/000112802

[B44] Adamian L Liang J Methods for analyzing deep sequencing expression data: constructing the human and mouse promoterome with deepCAGE data Genome Res 2009 14 R79 10.1186/gb-2009-10-7-r79 PMC2728533 19624849

[B45] Liu X Somel M Tang L Yan Z Jiang X He L Oleksiak A Zhang Y Li N Hu Y Chen W Qiu Z Paabo S Khaitovich P Extension of cortical synaptic development distinguishes humans from chimpanzees and macaques Genome Res 2012 14 611 622 10.1101/gr.127324.111 22300767 PMC3317144

[B46] Bullard JH Purdom E Hansen KD Dudoit S Evaluation of statistical methods for normalization and differential expression in mRNA-Seq experiments BMC Bioinformatics 2010 14 10.1186/1471-2105-11-94 PMC2838869 20167110

[B47] Bishop CM Pattern Recognition and Machine Learning 2009 New York: Springer Science

[B48] Szczurek E Biecek P Tiuryn J Vingron M Introducing knowledge into differential expression analysis J Comput Biol 2010 14 953 967 10.1089/cmb.2010.0034 20726790 PMC3122906

[B49] Pique-Regi R Deqner J Pai A Gaffney D Gilad Y Pritchard J Accurate inference of transcription factor binding from DNA sequence and chromatin accessibility data Genome Res 2011 14 447 455 10.1101/gr.112623.110 21106904 PMC3044858

[B50] Li W Godzik A Cd-hit: a fast program for clustering and comparing large sets of protein or nucleotide sequences Bioinformatics 2006 14 1658 1659 10.1093/bioinformatics/btl158 16731699

[B51] Quinlan AR Hall IM BEDTools: a flexible suite of utilities for comparing genomic features Bioinformatics 2010 14 841 842 10.1093/bioinformatics/btq033 20110278 PMC2832824

[B52] Roider H Kanhere A Manke T Vingron M Predicting transcription factor affinities to DNA from a biophysical model Bioinformatics 2007 14 134 141 17098775 10.1093/bioinformatics/btl565

[B53] Manke T Roider H Vingron M Statistical modeling of transcription factor binding affinities predicts regulatory interactions Plos Comput Biol 2008 14 e1000039 10.1371/journal.pcbi.1000039 18369429 PMC2266803

[B54] Edgar RC MUSCLE: multiple sequence alignment with high accuracy and high throughput Nucleic Acid Res 2004 14 1792 1797 15034147 10.1093/nar/gkh340 PMC390337

