# Supplementary material for: PROmiRNA: a new miRNA promoter recognition method uncovers the complex regulation of intronic miRNAs
Source: Genome Biol. 2013 Aug 16;14(8):R84. doi: 10.1186/gb-2013-14-8-r84 (PMC4053815; doi:10.1186/gb-2013-14-8-r84)
Supplement: Additional file 1 — This file contains a detailed description of the data set used, the method and additional results. [file gb-2013-14-8-r84-S1.PDF]

Supplementary material for: A new miRNA promoter recognition method uncovers the complex regulation of intronic miRNAs

Annalisa Marsico*[1] and Matthew Huska[1] and Julia Lasserre[1] and Haiyang Hu[2] and Dubravka Vucicevic[1] and Anne Musahl[1] and Ulf Andersson Orom[1] and Martin Vingron[1]

[1]Max Planck Institute for Molecular Genetics, Ihnestrasse 63-73, 14195 Berlin, Germany
[2]Partner Institute for Computational Biology, Shanghai Institutes for Biological Sciences, Chinese Academy of Sciences, 320 Yue Yang Road, 200031 Shanghai, China

Email: Annalisa Marsico*- marsico@molgen.mpg.de; Matthew Huska - huska@huska@molgen.mpg.de; Julia Lasserre - lasserre@molgen.mpg.de; Haiyang Hu - oceanhu@126.com; Dubravka Vucicevic - vucicevi@molgen.mpg.de; Anne Musahl - musahl@molgen.mpg.de; Ulf Andersson Orom - oerom@molgen.mpg.de; Martin Vingron - vingron@molgen.mpg.de;

*Corresponding author

Contents

1 Introduction **2**

1.1 miRNA promoter recognition 2

1.2 Overview 4

2 Data sources and Processing **5**

2.1 CAGE Data 5

2.2 Data for the prior 7

2.3 Data for TFBs analysis 7

2.3.1 TRAP analysis 7

2.3.2 Matrix scan analysis 8

2.4 PolII ChIP-seq data from ENCODE 8

2.5 RNA-Seq Data from the Human Body Map Data 2.0 Project (GEO: GSE30611) 10

3 The mirCAGE model in detail **10**

3.1 The model 10

3.2 EM algorithm for parameter estimation . . . 11

3.2.1 E step . . . 12

3.2.2 M step . . . 12

3.3 Estimation of the initial values of the model parameters . . . 12

3.4 Estimation of the beta parameters . . . 13

3.5 EM convergence criteria . . . 13

4 Impact of read count distributions on the PROmiRNA model 13

4.1 Performance of the PROmiRNA model compared to a 'simple model' . . . 13

4.2 Read count ditributions . . . 14

5 MiRNA promoter transcription factor binding site analysis 15

6 Tissue enrichment of intronic promoters 17

7 Comparison with ENCODE-generated CAGE TSSs 18

8 Description of the other supplementary files 19

8.1 Additional file 2 . . . 19

8.2 Additional file 3 . . . 20

1 Introduction

1.1 miRNA promoter recognition

The understanding of microRNA transcription is crucial for elucidating in detail their biogenesis, identify their regulators, as well as the role they may play in complex regulatory networks. There are mainly two classes of miRNAs : those embedded in introns or exons of annotated genes (intragenic miRNAs) and those located in intergenic regions of the genome (intergenic miRNAs). It is widely accepted that intronic miRNAs can, either be cotranscribed with their host gene [1] or have an independent promoter [2], while intergenic miRNAs can derive from transcripts of up to 50 kb in length, referred to as pri-miRNAs [3]. Although the canonical view of miRNA processing is not universal for all miRNAs and evidence for post-transcriptional control has been accumulating [4], in mammalian systems the linear miRNA processing pathway is mainly the one depicted in Figure S1. Primary miRNA transcripts (pri-miRNAs) may contain multiple miRNA stem loops, many of them are polyadenylated, capped and occasionally spliced - all hallmarks of Polymerase II transcription. However, it has been found that some miRNAs are transcribed by Polymerase III. In the

canonical pathway, pri-miRNA processing happens in two steps: in the first step, which happens in the nucleus, the enzyme Drosha cleaves the pri-miRNA transcript into a 70-nucleotide precursor hairpin (pre-miRNA). The second step consists in cleavage by Dicer, after the pre-miRNA is exported to the cytoplasm. This yelds a 20-bp miRNA/miRNA duplex. Usually one strand of this duplex, the mature miRNA, associates with several members of the Argonaute protein family to form the miRNA-induced silencing complex (miRISC). This then binds to target mRNAs and induces their repression or degradation. The miRNA strand is usually released and degraded [3].

Due to their transient nature, i.e. fast Drosha cleavage in the nucleus, pri-miRNAs, and more specifically miRNA TSSs, are hard to identify. Therefore, there is a huge need for miRNA promoter recognition algorithms in order to locate the miRNA TSSs genome-wide, and ultimately aim at identifying the miRNA core promtoers and the upstream regulatory elements. [3].

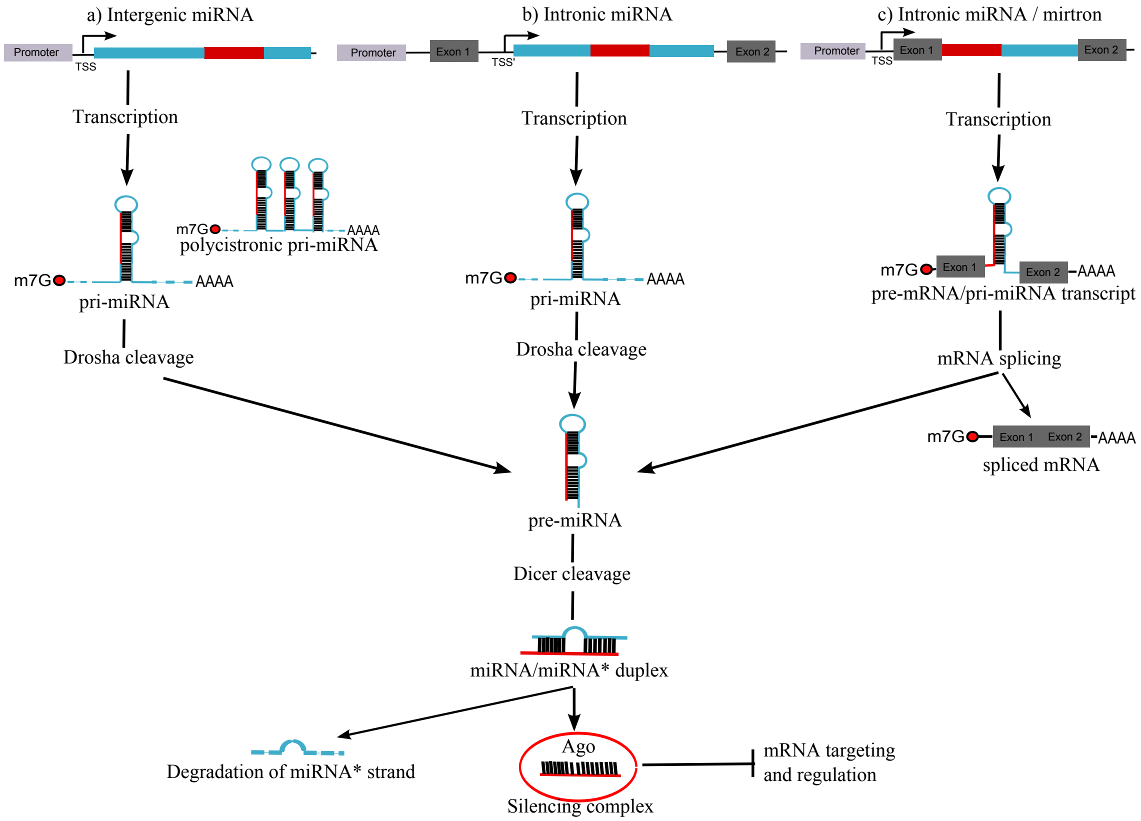

Figure S1: **miRNA biogenesis canonical pathway.** microRNAs can be found in the genome as independent gene units (a), i.e. intergenic miRNAs, or be embedded in introns of protein/lncRNA genes called host genes (b,c). When contained inside a gene, the miRNA may use the TSS of the host gene and be co-transcribed with it or its own TSS (b), like the intergenic miRNAs, generating a primary transcript called pri-miRNA. The pri-miRNA can contain multiple miRNA stem loops, be several kb long and it usually contains a cap at the 5' end and a poly(A). In the first step of the biogenesis pathway, the pri-miRNA is cut by the Drosha enyzme to produce a 70 bp long precursor hairpin (pre-miRNA), which is exported to the cytoplasm. Some pre-miRNAs are produced by very short introns (mirtrons) as a result of splicing and debranching, thereby bypassing the Drosha step (c). In either case, the second step consists in cleavage by Dicer in the cytoplasm, which yelds a 20 bp miRNA/miRNA* duplex. Usually one strand of this duplex, the mature miRNA, associates with the miRNA-induced silencing complex (miRISC), which then binds to target genes and induces their translational repression or degradation, and the miRNA* is degraded.

1.2 Overview

Our promoter recognition algorithm applies a semi-supervised mixture model approach to classify candidate genomic regions as putative miRNA promoters. It starts by identifying a set of genomic regions enriched in CAGE tags upstream of annotated mature miRNAs, and tries to classify them into putative promoters and background sequences. These two classes of genomic regions have different distributions of the log of tag counts, and can be fitted by a mixture model. We use a EM (Expectation Maximization) algorithm to learn the parameters of the distribution and maximize the likelihood of the data. Other relevant information,

such as CpG content, conservation across vertebrates, TATA box binding affinity and distance from the mature miRNA are computed for both classes and included in the model as the *prior* probability of a certain region to be a promoter or a background region. Random intergenic regions are also used in the model as labeled examples (posterior probability equal to 1.0 for the background class), in order to make our learning algorithm semi-supervised. The algorithm reports a set of candidate regions with higher posterior probability of belonging to the promoter than to the backgorund class.

2 Data sources and Processing

2.1 CAGE Data

The CAGE data for the human genome (hg18 assembly) are downloaded from the public resource of FANTOM4 project (http://fantom.gsc.riken.jp/4/download/Tables/human/CAGE [5]). We re-map the raw data, corresponding to the genomic positions of mapped tags, on the hg19 human assembly using the LiftOver tool in the UCSC Genome Browser. We keep only tags that map uniquely to the genome for further analysis. The 33 samples used in this study (we consider replicates only once) are listed in Table S1, together with the names of the RNA libraries in each sample, their description and the number of uniquely mapped reads.

**RNA Libraries**	**tissue/cells**	**uniquely mapped reads to hg19**
HAM,HAN,HAO.HAP	cerebrum	89542
HAW, HAX, HAY, HAZ	renal artery, ureter, urinary bladder	41,036
HAJ, HAU, HAV	kidney, kidney malignancy	76,952
HAD, HAE, HAF, HAg, HAH	small intestine, rectum malignancy, rectum, cecum malignancy, cecum	111,524
HAK, HAL, HAQ, HAR, HAS, HAT	stomach, liver, large intestine, large intestine malignancy	101,807
HAI	kidney	25,653
HBA, HBB, HBC	prostate gland, mammary gland, epididymis	22655
HBY	HB-6062 Hep G2	213,042
HBM	CRL-2429 CCD-1112SK	207,473
HBP, HBQ	liver, liver malignancy	78,734
HBD, HBE, HBF	adipose, adipose DHT 9 days, adipose TNFa 2 days	80,835
HBJ, HBK, HBL	adipose, adipose DHT 8 days, adipose TNF2 2 days	80,835
HBR, HBS	pancreas, pancreas malignancy	48,886
HBU	thymus	17,660
HAA, HAB, HAC	untreated cells, VIT3 96 hours, retinoid acid 96 hours	202,363
HBZ, HCA, HCF, HCG	blood, lung	244,702
HCB, HCC, HCD, HCE, HCH	adrenal gland, colon, skin	297,364
HCI, HCJ, HCO	brain, cerebellum	245,917
HCX, HCY, HDA	brain, testis, thymus	171,147
HDC, HDD, HDE	embryo	113,541
HDO, HDP, HDQ	brain	167,800
HEM, HEN, HEP, HEQ	bone marrow, liver	266,317
HDR, HDS, HDt, HDU, HDV, HDW	lung, liver	184,853
HDI, HDJ, HDK, HEA, HEB, HEC	heart, muscle	144,977
HCR, HCS, HCT, HCV	brain, frontal lobe, occipital lobe, parietal lobe	169,128
HCK, HCL, HCM	colon, spleen	95,132
HDF, HDG, HDH	embryo	118,982
HES	mammary gland	35,905
HER	breast	32,905
THP-1	THP-1 8hr, THP-1 12hr, THP-1 18hr, THP-1 24hr	998,610
HFG, HFH, HFI, HFJ, HEK, HEX	PMA 1hr, PMA 4hr, PMA 12hr, PMA 24hr, PMA 96hr, PMA control	1,460,782
HFL, HFM	CD4+, control	561,584
HFG, HFH, HFI, HFJ, HEK, HEX	PMA 1hr, PMA 4hr, PMA 12hr, PMA 24hr, PMA 96hr, PMA control	1,460,782

Table S1: **Summary of the FANTOM4 deepCAGE libraries used in this study**

2.2 Data for the prior

The genomic data used for calculating the different terms of the prior probability in our model are listed below:

1. CpG content of the 1000 bp promoter region around the identified TSS, computed based on Equation 7 (main text, Material and Methods)

2. Average PhastCons conservation score of the promoter region across vertebrates on the 46-way alignment (http://hgdownload.cse.ucsc.edu/goldenPath/hg19/phastCons46way/vertebrate/)

3. TATA box affinity. We derive the regulatory motif for a TATA box, described as a position-specific scoring matrix (pscm), from the Jaspar database (http://jaspar.genereg.net/, Jaspar ID: MA0108.2). We convert the pscm matrix to a position-specific mismatch energy matrix (psem), as described in [6] and calculate the total TRAP affinity for the 1000 bp long promoters with a speudocount of 0.25 and average GC content parameter set to 0.5 (see [7] for details).

4. distance d in base pairs of the TSS from the annotated miRNA precursor (annotation taken from miRBase release 19). We calculate the miRNA proximity score according to the following formula:

$$\text{mirna\_proximity} = \left(1 + \frac{d}{1000}\right)^{-1} \tag{1}$$

2.3 Data for TFBs analysis

2.3.1 TRAP analysis

TRAP (Transcription Factor Affinity Prediction) applies a physical model to predict the relative binding affinities of TFs to DNA regulatory regions, by integrating the contribution from individual strong and weak sites. A detailed description of the physical model is given in [6]. Unlike hit-based method for TFBs detection, TRAP does not search for 'hits' of a certain factor in a sequence, based on an arbitrary score threshold for match/no match. Instead, it provides a quantificaiton of the TF binding affinity for a certain region, by taking into account all possible binding sites, even the weak ones, in that region. Indeed, it has been shown that sometimes the binary view of TFBSs math/no match might not reflect the real biological situation where several weak binding sites along a certain sequence may play a strong role in regulation [6]. We download the miRNA promoter sequences, defined as +/- 500 bp around the identified TSSs, from the UCSC. We transform the 130 core vertebrate matrices from the JASPAR database (http://jaspar.genereg.net) into position-specific mismatch energy matrices (psem) and add a pseudo-count of 0.25, as described in [6].

TRAP is used on the transformed matrices to compute transcripion factor binding site affinity values of miRNA promoter sequences with default parameters.

TF normalized affinities and associated p-values are calculated as described in [8], by comparing the observed TF affinity to the affinity distribution of the background sequences. We use the 1000 bp regions upstream the TSS of all annotated genes as background sequences. P-values for individual miRNA promoter sequences are combined by the Fisher's method, as described in [7]. The Benjamin-Hochberg method is used to correct the combined p-values.

2.3.2 Matrix scan analysis

We use a command-line version of the pattern matching program Matrix Scan [9], kindly provided from the authors, to search for singificant TFBs hits in miRNA promoter sequences, using the 130 core vertebrate position-specific scoring matrices from the Jaspar database (http://jaspar.genereg.net). We used a background model of Markov order two, estimated from the input sequences, to compute the significance of each hit, and a p-value threshold of 0.001 for filtering out non-signifcant hits.

2.4 PolII ChIP-seq data from ENCODE

For evaluating the precision of our miRNA promoter annotation model we used the pooled PolII ChIP-seq data from the ENCODE project on several cell lines. We computed the overlap of our promoters with the alredy computed peaks from all libraries. The data were downloaded from http://genome.ucsc.edu/cgi-bin/hgFileUi?db=hg19&g=wgEncodeHaibTfbs. The accession ids and total number of PolII peaks for each library are reported in Table S2.

Cell line	UCSC Accession	GEO Accession	num of peaks
A549	wgEncodeEH001494	GSM803361	26042
A549	wgEncodeEH001494	GSM803361	40596
A549	wgEncodeEH001493	GSM803360	37374
A594	wgEncodeEH001493	GSM803360	52728
ECC_1	wgEncodeEH001572	GSM803536	20650
ECC_1	wgEncodeEH001572	GSM803536	35977
GM12878	wgEncodeEH001463	GSM803355	51412
GM12878	wgEncodeEH001463	GSM803355	58308
GM12878	wgEncodeEH001517	GSM803485	54813
GM12878	wgEncodeEH001517	GSM803485	57209
GM12891	gEncodeEH001522	GSM803399	42106
GM12891	gEncodeEH001522	GSM803399	37201
GM12891	wgEncodeEH001523	GSM803400	32698
GM12891	wgEncodeEH001523	GSM803400	32894
GM12892	wgEncodeEH001511	GSM803487	39443
GM12892	wgEncodeEH001511	GSM803487	38312
GM12892	wgEncodeEH001512	GSM803490	37493
GM12892	wgEncodeEH001512	GSM803490	29054
H1-hESC	wgEncodeEH001499	GSM803366	42559
H1-hESC	wgEncodeEH001499	GSM803366	91979
H1-hESC	wgEncodeEH001514	GSM803484	32797
H1-hESC	wgEncodeEH001514	GSM803484	41908
HCT-116	wgEncodeEH001627	GSM803474	34980
HCT-116	wgEncodeEH001627	GSM803474	42690
HUVEC	wgEncodeEH002297	GSM1010830	34174
HUVEC	wgEncodeEH002297	GSM1010830	33535
HUVEC	wgEncodeEH002298	GSM1010824	24094
HUVEC	wgEncodeEH002298	GSM1010824	21689
HeLa-S3	wgEncodeEH001474	GSM803533	37384
HeLa-S3	wgEncodeEH001474	GSM803533	54266
HepG2	wgEncodeEH001550	GSM803368	36391
HepG2	wgEncodeEH001550	GSM803368	35183
HepG2	wgEncodeEH002278	GSM1010821	25570
HepG2	wgEncodeEH002278	GSM1010821	34559
K562	wgEncodeEH001633	GSM803410	25869
K562	wgEncodeEH001633	GSM803410	50265
K562	wgEncodeEH001581	GSM803443	48049
K562	wgEncodeEH001581	GSM803443	57411
PANC-1	wgEncodeEH002265	GSM1010788	27863
PFSK-1	wgEncodeEH002272	GSM1010819	38191
PFSK-1	wgEncodeEH002272	GSM1010819	18237
SK-N-MC	wgEncodeEH002272	GSM1010819	32485
SK-N-MC	wgEncodeEH002272	GSM1010819	29908
SK-N-SH	wgEncodeEH002270	GSM1010817	35606
SK-N-SH	wgEncodeEH002270	GSM1010817	65287
U87	wgEncodeEH001674	GSM803459	25249
U87	wgEncodeEH001674	GSM803459	34076

Table S2: ENCODE PolII ChiP-seq experiments used for miRNA promoter validation and number of peaks for each library

2.5 RNA-Seq Data from the Human Body Map Data 2.0 Project (GEO: GSE30611)

For the validation of full-length pri-miRNA transcripts originating from the identified promoters we use the RNA-Seq data from the Human Body Map Data 2.0 Project (GEO: GSE30611). We download the raw data for the 16 libraries of 100 bp stranded RNA, each corresponding to a 16 tissue mixture, from SRA (http://www.ncbi.nlm.nih.gov/sra, ID: ERP000546). For each library we map the reads on the hg19 human genome asembly with GSNAP [10], and the number of uniquely mapped reads for each library are reported in the Table S3.

**Flow Cell A (FCA)**	
**lane**	**# uniquely mapped reads**
lane 1	51,645,998
lane 2	52,416,285
lane 3	50,015,881
lane 4	51,280,067
lane 5	48,445,350
lane 6	47887974
lane 7	38,125,628
lane 8	42,574,001
**Flow Cell B (FCB)**	
lane 1	53,654,578
lane 2	52,494,988
lane 3	55,891,493
lane 4	55,983,385
lane 5	37,885,315
lane 6	37,566,067
lane 7	37,514,029
lane 8	37,727,253

Table S3: Summary of mapped reads for the RNA-Seq libraries from the Human Body Map 2.0 Project

3 The mirCAGE model in detail

3.1 The model

MirCAGE uses a partially-supervised mixture modeling methodology for miRNA promoter recognition, which incorporates knowledge about promoter features into TSS predictions from high-throughput sequencing data (CAGE data). Given a certain genomic region i associated to X_i (quantile-normalized) CAGE tag counts:

$$P(X_i) = \sum_k P(Z_{ik} = 1|G_i)P(X_i|Z_{ik} = 1) \tag{2}$$

where X_i can come from two underlying distributions that form the mixture model ($k = \{1, 2\}$), and correspond to real promoters ($Z_{i1} = 1$) and background noise regions ($Z_{i2} = 1$). G_i represents some prior

information about region i. The model is partially supervised because, for each candidate promoter region, we model the prior probability of being a real promoter $\pi_{ik} = P(Z_{ik} = 1|G_i)$ on the basis of calculated genomic features, such as CpG content, conservation score, TATA box affinity and miRNA proximity, according to the belief-based mixture model proposed in [11] for gene expression analysis. The prior probability is modeled using a logistic function:

$$\pi_{i1} = \frac{1}{1 + e^{-y_i}} \ ; \ \pi_{i2} = 1 - \pi_{i1} \tag{3}$$

$$y_i = \beta_0 + \beta_1 \cdot CpG_i + \beta_2 \cdot \text{cons}_i + \beta 3 \cdot \text{TATA}_i + \beta_4 \cdot \text{mirna\_proximity}_i \tag{4}$$

The prior probability π_{i1} is different for each region i and represents the *belief* for the region to be a real promoter. We model the two tag count probability distributions $P(X_i|Z_{ik} = 1)$ for $k = \{1, 2\}$, which distinguish promoters from background, by means of two inverse Gaussian distributions of parameters μ_k and λ_k:

$$P(X_i|Z_{ik} = 1) = \left(\frac{\lambda_k}{2\pi \cdot X_i^3}\right)^{1/2} \cdot \exp\left(\frac{-\lambda_k (X_i - \mu_k)^2}{2\mu_k^2 X_i}\right) \tag{5}$$

More generally, given a set N of candidate promoter regions, the probability distribution of the data $\mathbf{X} = \{X_i\}$ is given from the following:

$$p(\mathbf{X}) = \prod_i \left(\sum_k \pi_{ik} \cdot P_k(X_i|\mu_k, \lambda_k)\right) \tag{6}$$

where $P_k(X_i|\mu_k, \lambda_k)$ is the tag count (the logarithm of the tag counts) probability distribution depening on the μ_k and λ_k parameters.

3.2 EM algorithm for parameter estimation

The EM algorithm is a method for finding a solution to a maximum likelihood problem, such as the estimation of the unknown parameters of a model depending on the latent variables Z_k [12]. Altough it is usually applied to Gaussian mixture models, in our case we apply it to determine the maximum likelihood estimates of the μ_k and λ_k parameters of a mixture of Inverse Gaussian distributions. In addition, in our model, instead of learning the mixing coefficients or prior probabilities π_{ik} in the EM procedure, we fix their values in advance, estimating them by means of logistic regression, as described in the next paragraph. We choose some initial values for the μ_k and λ_k parameters (as described later) and we alternate between the E (Expectation) step, where we estimate the expectation of the log-likelihood of the data using the current parameter estimates, and the M (Maximization) step, where we re-estimate the parameter values. We iterate between these two

steps till convergence. In detail, the log-likelihood of the data is given from:

$$\log P(\mathbf{X}|\pi, \mu, \lambda) = \sum_i \log \left\{ \sum_k \pi_{ik} P_k(X_i|\mu_k, \lambda_k) \right\} \tag{7}$$

where P_k is the Inverse Gaussian probability density function.

3.2.1 E step

Calculating the expectation of the log-likelihood function is equivalent to determining the distributions of the Z_k, the latent variables, given $\mathbf{X}$. This is given by the Bayes formula:

$$\gamma_i(Z_{ik}) \equiv p(Z_{ik} = 1|X_i) = \frac{p(Z_{ik} = 1)p(X_i|Z_{ik} = 1)}{\sum_k p(Z_{ik} = 1)p(X_i|Z_{ik} = 1)} = \frac{\pi_{ik} P_k(X_i|\mu_k, \lambda_k)}{\sum_k \pi_{ik} P_k(X_i|\mu_k, \lambda_k)} \tag{8}$$

For $k = 1$ (promoter class), $p(Z_{ik} = 1|X_i)$ represents the probability of candidate region i to be a real promoter, given the tag count observaiton X_i, while for $k = 2$ (backround region class) is the probability of being noise. $P(Z_{ik} = 1) \equiv \pi_{ik}$ represents the prior probability to belong to the promoter class when $k = 1$, and to the background class if $k = 2$. The denominator in Equation 8 is the sum of the conditional probability of the data over all the components k. $\gamma_i(Z_{ik})$ is then the posterior probability once we have observed X_i.

3.2.2 M step

It consists in the maximization of the log-likelihood function with respect to the distribution parameters

$$\mu_k = \mathrm{argmax}_{\mu_k} E[\log P_k(\mathbf{X}|\pi_k, \mu_k, \lambda_k)] \tag{9}$$

$$\lambda_k = \mathrm{argmax}_{\lambda_k} E[\log P_k(\mathbf{X}|\pi_k, \mu_k, \lambda_k)] \tag{10}$$

By solving these equations with respect to μ_k and λ_k we obtain:

$$\mu_k = \frac{\sum_i \gamma_i(Z_k) X_i}{\sum_i \gamma_i(Z_k)} \tag{11}$$

$$\lambda_k = \mu_k^2 \cdot \frac{\sum_i \gamma_i(Z_k)}{\sum_i \gamma_i(Z_k) \cdot \frac{(X_i - \mu_k)^2}{X_i}} \tag{12}$$

3.3 Estimation of the initial values of the model parameters

The EM algorithm estimates the model components starting from some initial values of the paramters μ_k and λ_k. The definition of appropriate initial parameters is crucial for the EM algorithm to work properly and there are various ways of defining them. Here we compute initial values for μ_k and λ_k from a limited

set of examples using the maximum likelihood estimations in equations 11 and 12. We define an *ad-hoc* set of exact examples including 1000 promoters and 1000 background regions. We extract background regions randomly from intergenic and intronic genomic regions and we choose the real promoters randomly from those candidate tag-enriched regions found across more than 10 FANTOM4 libraries.

3.4 Estimation of the beta parameters

The parameters β_0,β_1,β_2,β_3 and β_4 of the prior probability (Equation 4) are not learned by the EM algorithm, as there is no closed-form solution that maximizes the log-likelihood of the data. For this reason we estimate the β parameter values in advance and leave them fixed while learning the inverse Gaussian distribution parameters during the EM iterations. The β parameters are estimated from the set of 'exact' background regions and promoter examples defined in the previous paragraph, by means of a logistic function (*glm* R function). The values of the β paramters after fitting the logistic regression model represent the relative contributions of each genomic feature to the prior.

3.5 EM convergence criteria

At every iteration j of the EM algorithm we compute the difference between the total log-likelihood of the data $log(P(\mathbf{X}))_j$ at j and the log-likelihood at the previous iteration $j-1$. The convergence criteria is satisfied (i.e. the algorithm stops) when:

$$\log P(\mathbf{X})_j - \log(\mathbf{X})_{j-1} < 0.1 \tag{13}$$

4 Impact of read count distributions on the PROmiRNA model

4.1 Performance of the PROmiRNA model compared to a 'simple model'

The use of the prior probability in our model is justified from the fact that low read count regions not necessarily correspond to noise but might represent lowly expressed miRNA promoters. In addition, low read count reigons might also indicate fast processing of the primary transcript but still correspond to the true TSS location. For this reason, a mixture model based only on read count distributions would be able to retrieve the highly expressed promoters but would wrongly classify a big proportion of lowly expressed promoters. In order to demonstrate this, we started with our set of candidate regions upstream of miRNAs hit by CAGE tags and, based on PolII data, we computed the performance of:

- a 'simple' model which uses only read counts but not the prior probability

- a model which ignores the read count intensities and only uses the prior probability
- the complete PROmiRNA model, which uses both cage tags and prior information

Figure S2 shows that a simple model based on number of cage tags only reaches a very low precision for all three promoter classes (hostgene, intergenic and intronic) This clearly indicates that the tag count criteria alone would not separate properly miRNA promoters from non-promoters. On the other hand, when using only the prior probability on the regions hit by at least one tag, the performance of the method is already good, but increases further when a combination of both cage tag and other features is modeled in the complete model.

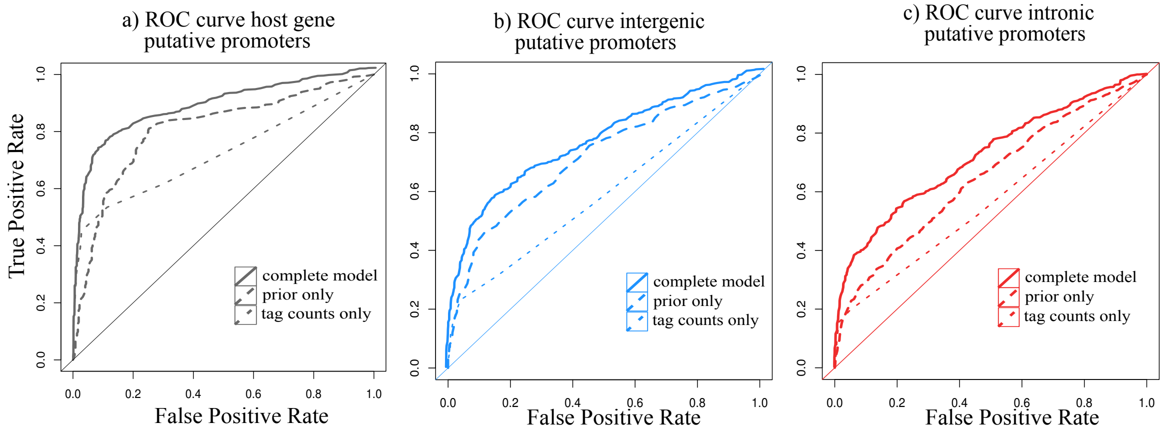

Figure S2: Performance of the PROmiRNA *complete* model versus the performance of a *simple* model and to a model based on prior only. The performance, based on the comparison with PolII data, is show separately for three promoter classes: hostgene, intergenic and intronic miRNA promoters.

4.2 Read count ditributions

The tag counts for putative TSSs, defined as described in Material and Methods in the main text, from all the 33 RNA libraries have been pooled together, and the distribution of their logarithm has been plotted (black curve, Figure S3a). We added a pseudocount of 2.0 to each tag count value before computing the logarithm. The red and blue distributions, superimposed to the empirical tag count distirbution, represent the theoretical distribution for putative TSS and background regions, respectively, given the parameters inferred from EM algorithm when modeling the data with the 'simple' model described above (without including the prior probability into the model). The values of the parameters are $\mu_1 = 1.61$, $\lambda_1 = 13.38$ and $\mu_2 = 0.94$, $\lambda_2 = 10.8$ for promoters and background regions, respectively. Figure S3b shows the real disitrbution of cage tags for predicted promoters (red curve) and background regions (blue curves) when

the regions are classified according to a complete model, including both tag counts and the prior probability calculated from the computed promoter features (as described in the previous paragraph). The plots show that low-count regions, which would be excluded from the promoter class from the ‘simple’ model are putative promoters, which are picked up from the ’complete’ model, when taking the prior into account. This clearly shows that the two modes are not simply dictated by the number of cage tags, but that some other features (i.e. the prior) contribute to the separation of the blue class from the red class.

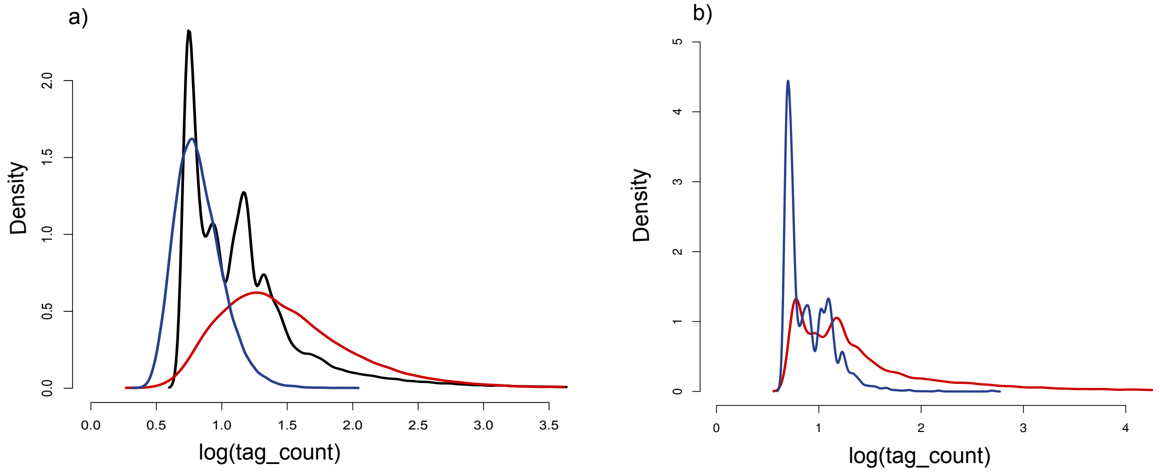

Figure S3: **a)** Distribution of tag counts in promoters and background regions according to the ’simple’ model. **b)** Distribution of tag counts in predicted promoters and background regions according to the ’complete’ model.

5 MiRNA promoter transcription factor binding site analysis

Table S4 lists the transcription factors with highest affinity, according to the TRAP prediction, for the three different classes of identified miRNA promoters: host gene, intergenic and intronic. Each column correponds to a different promoter class, and in each column the factors are ranked accoring to the the TRAP p-value (not shown), which indicates the significance of the enrichment of a certain factor in that class. We only report statistically signifcant enriched factors (corrected $p < 0.01$). The transcription factors shown in red are those factors which are also found from Matrix Scan to be among the 50 top factors with significant binding sites in that particular set of sequences.

Top enriched TFBs		
Host gene	Intergenic	Intronic
SP1	EBF1	Pdx1
Egr1	RREB1	Foxa2
PLAG1	PLAG1	Sox5
Klf4	NF-kappaB	TBP
Pax5	INSM1	Prrx2
Mycn	USF1	ARID3A
NFKB1	MZF1	FOXO3
E2FI	Pdx1	Gata1
Myc	REL SP1	SRY
MIZF	Stat3	FOXI1
Zfx	Egr1	Nkx3-1
RREB1	Mycn	FOXA1
GABPA	Foxa2	Foxq1
Arnt	Arnt	HOXA5
REST	NFATC2	NFATC2
zfp423	RELA	MEF2A
Tcfcp211	Pax4	HNF1B
NHLHI	STAT1	NR3CI
Mafb	Klf4	Pou5f1
USFI	RORA1	NFIL3
ELK4	FOXI1	FOXF2
Myf	CTCF	FOXD1
EBFI	ESR2	HNFIA
Myb	Sox5	SOX10
NFYA	HNF4A	Nkx2-5
RELA	TFAP2A	Lhx3
ESRI	FOXF2	IRF1
Max	PPARG	FOXL1
TP53	TBP	RREB1
	FOXO3	RORA1
	SOX10	AP1
	TP53	Pax4
		TEAD1
		IRF2
		PBX1
		Foxd3
		Sox9
		STAT1
		Esrrb
		SRF
		ELF5
		NFE2L2
		NR4A2
		RUNX1
		NR2FI
		SPI1
		SPZ1

Table S4: **Top enriched Transcription Factor Binding Sites (TFBs)**

6 Tissue enrichment of intronic promoters

In order to check if intronic miRNA promoters are preferentially used in certain tissues and depleted in others, we compute the enrichment of intronic promoters in each FANTOM library. The results are shown in Figure S4. The odd ratio on the y-axis indicates the fraction of intronic promoters in a certain library, divided by the fraction of non-intronic promoters in that library. Significant enrichment or depletion of intronic promoters, according to a Fisher exact test, is indicated by a star symbol ($p\_value <= 0.0015$, Bonferroni correction). We find that intronic promoters are significantly enriched in brain, thymus, lung and ambryo, while being depleted in breast, mammary gland, bone marrow, blood, T cells, monocytic cells and HepG2.

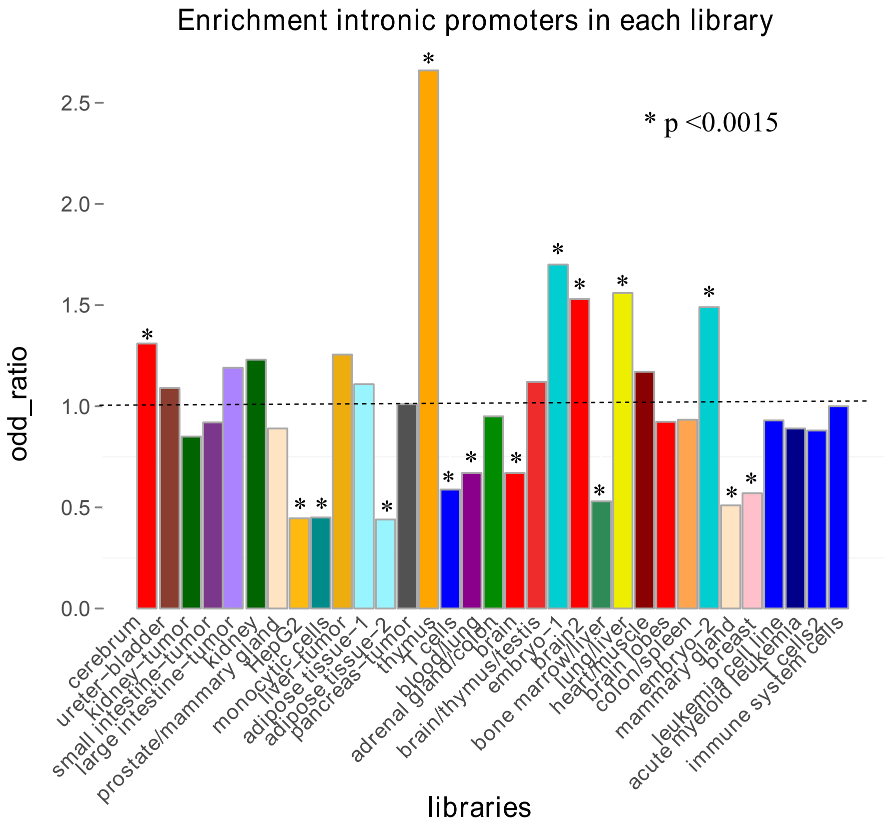

Figure S4: **Enrichment or depletion of intronic promoters in invidual CAGE libraries**

7 Comparison with ENCODE-generated CAGE TSSs

While this manuscript was in preparation, new CAGE data from the RIKEN Institute were released inside the ENCODE project. Although no specific annotation for miRNA promoters is provided from these tracks, we checked our miRNA promoters against the ENCODE-generated CAGE TSSs. The data for comparison, corresponding to TSS regions surrounding CAGE clusters, and based on Hidden Markov model predictions, were downloaded from the following link http://genome.ucsc.edu/cgi-bin/hgFileUi?db=hg19&g=wgEncodeRikenCage and pooled together. The ENCODE TSS predictor assigns a posterior probability to a certain candidate region to be a real promoter based on a Hidden Markov Model trained on the genomic sequences surrounding CAGE tag clusters. Therefore, we evaluated the number of our predicted miRNA promoters that overlapped with the ENCODE CAGE TSSs at different cutoffs of their posterior probability (Figure S5). The comparison indicates that almost 100% of our predicted miRNA host gene promoters are confirmed from the ENCODE TSSs based on the new CAGE data. On the other hand, while the overlap for intergenic miRNA does not depend from the cutoff and is about 60%, the overlap for intronic promoters significantly drops as soon as the HMM cutoff is increasesd. This is could be due to reasons: first, the RNA libraries used for generating the new ENCODE CAGE data are different from the ones used in the FANTOM4 project, and the location and expression of tissue-specific promoters might differ from one library to the other. Second, The Hidden Markov Model used in ENCODE to annotate TSSs discards transcriptional start sites with less than 10 reads in all libraries. As miRNA promoters in general, and intronic promoters in particular, have a smaller number of associated tags with respect to host gene promoters, due to quick pri-miRNA processing, a threshold of 10 reads might miss many of them.

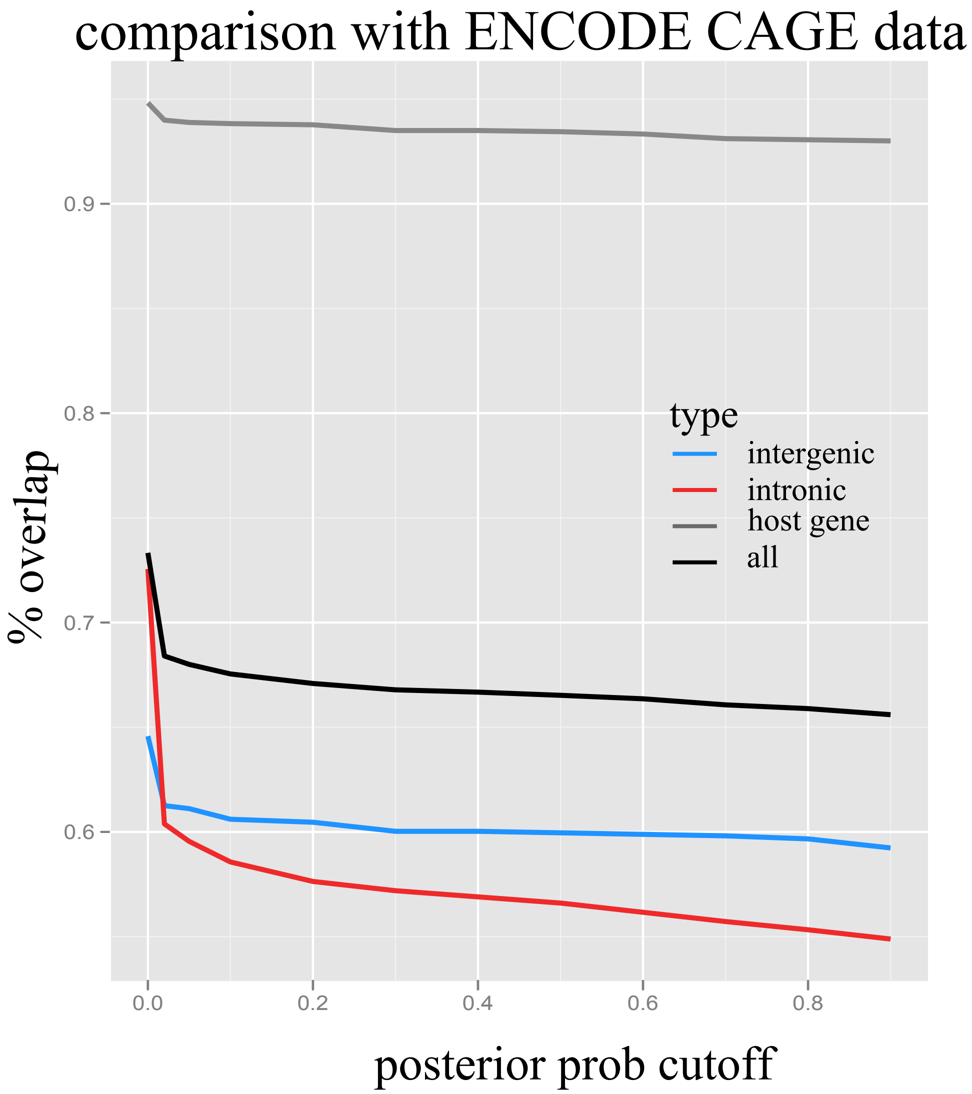

Figure S5: **Comparison with ENCODE CAGE data**

8 Description of the other supplementary files

8.1 Additional file 2

This table reports all predicted promoters for the annotated miRNAs (miRBase v18). The columns correspond to the following attributes:

miRNA name of the miRNA corresponding to the predicted TSS

chrom chromosome where the miRNA is located

miRNA_start start position of the miRNA precursor

miRNA_stop stop position of the miRNA precursor

strand strand

tss_start start position of the TSS predicted from PROmiRNA

tss_stop stop position of the TSS predicted from PROmiRNA

width(bp) width of the predicted transcriptional start region

distance distance of the predicted TSS from the start of the miRNA precursor

type type of the predicted TSS (intergenic, host gene, intronic or hybrid)

normalized_tags number of quantile-normalized cage tags associated to the predicted TSS

prior_probability prior probability of the predicted TSS, computed from equation 5-6 (main mauscript)

promoter_probability promoter posterior probability of the predicted TSS, computed from equation 8 (main manuscript)

background_probability background posterior probability of the predicted TSS, computed from equation 8 (main manuscript)

CpG content CpG content of the 1000 bp-long region surrounding the predicted TSS

PhastCons score Conservation score of the 1000 bp-long region surrounding the predicted TSS, computed from the PhastCons ENCODE track

TATA box affinity TATA box total affinity of the 1000 bp-long region surrounding the predicted TSS, computed with the TRAP tool [7]

top_10_TRAP_factors The first 10 transcription factors with highest affinity for the 1000 bp-long region surrounding the predicted TSS, according to the TRAP analysis [7]

FANTOM4 libraries tissues in which the predicted TSS was espressed

miRNA age age of the miRNAs corresponding to the predicted TSS. 'v' indicates that the miRNA is conseved up to the vertebrates lineage; 'm' indicates that the miRNA is conseved up to the mammals lineage and 'p' indicates that the miRNA is conserved only in the primates lineage

8.2 Additional file 3

This table reports all predicted miRNA promoters, the comparison with other methods and the evaluation results **miRNA** name of the miRNA corresponding to the predicted TSS

chrom chromosome where the miRNA is located

TSS_start start position of the miRNA precursor

TSS_stop stop position of the miRNA precursor

strand strand

PolII_peak List of ENCODE libraries where a PolII peak overlapping with the predicted TSS was found

peak_start start postion of the PolII peak with largest overlap with the predicted TSS region

peak_stop stop postion of the PolII peak with largest overlap with the predicted TSS region

overlap overlap between the predicted TSS region and the PolII peak with largest overlap

coverage_RNA_seq fraction of read coverage for the putative pri-miRNA starting at the predicted TSS from RNA-seq data

start_Barski start position of the miRNA promoter predicted from Barski et al. [13] if any overlap with our predicted TSS could be found. If no corresponding promoters could be found form Barski's method, this field is equal to None.

stop_Barski stop position of the miRNA promoter predicted by Barski et al. [13] if any overlap with our predicted TSS could be found. If no corresponding promoters could be found form Barski's method, this field is equal to None.

start_Ozsolak start position of the miRNA promoter predicted by Ozsolak et al. [2] if any overlap with our predicted TSS could be found in a surrounding of 1000 bp. If no corresponding promoters could be found from Ozsolak's method, this field is equal to None.

stop_Ozsolak stop position of the miRNA promoter predicted by Ozsolak et al. [2] if any overlap with our predicted TSS could be found in a surrounding of 1000 bp. If no corresponding promoters could be found from Ozsolak's method, this field is equal to None.

CAGE_Encode it indicates if an overlapping annotated TSS region could be found in the ENCODE CAGE data

start_cage_cluster start position of the overlapping TSS region from the ENCODE CAGE predictions, if any

stop_cage_cluster stop position of the overlapping TSS region from the ENCODE CAGE predictions, if any

References

1. Baskerville S, Bartel DP: **Microarray profiling of microRNAs reveals frequent coexpression with neighboring miRNAs and host genes**. *RNA* 2005, **11**(3):241–247.

2. Ozsolak F, Poling LL, Wang Z, Liu H, Liu XS, Roeder RG, Zhang X, Song JS, Fisher DE: **Chromatin**

structure analyses identify miRNA promoters. *Gene Dev* 2008, **22**(22):3172–3183.

3. Krol J, Loedige I, Filipowicz W: **The widespread regulation of microRNA biogenesis, function and decay**. *Nat Rev Genet* 2010, **11**(9):597–610.

4. Winter J, jung S, Keller S, Gregory RI, Diederichs S: **Many roads to maturity: microRNA biogenesis pathways and their regulation**. *Nat Cell Biol* 2009, **11**(3):228–234.

5. Kawaji H, Severin J, Lizio M, Waterhouse A, Katayama S, Irvine KM, Hume DA, Forrest AR, Suzuki H, et al PC: **The FANTOM web resource: from mammalian transcriptional landscape to its dynamic regulation**. *Genome Biol* 2009, **10**(4):R40.

6. Roider H, Kanhere A, Manke T, Vingron M: **Predicting transcription factor affinities to DNA from a biophysical model**. *Bioinformatics* 2007, **32**(2):134–141.

7. Thomas-Chollier M, Hufton A, Heinig M, O'Keeffe S, Masri N, Roider H, Manke T, Vingron M: **Transcription factor binding predictions using TRAP for the analysis of ChIP-seq data and regulatory SNPs**. *Nat Protoc* 2011, **6**(12):1860–1869.

8. Manke T, Roider H, Vingron M: **Statistical modeling of transcription factor binding affinities predicts regulatory interactions**. *Plos Comput Biol* 2008, **4**(3):e1000039.

9. Turatsinze JV, Thomas-Chollier M, Defrance M, van Helden J: **Using RSAT to scan genome sequences for transcription factor binding sites and cis-regulatory modules**. *Nat Protoc* 2008, **3**(10):1578–1588.

10. Wu T, Nacu S: **Fast and SNP-tolerant detection of complex variants and splicing in short reads**. *Bioinformatics* 2010, **26**(7):873–881.

11. Szczurek E, Biecek P, Tiuryn J, Vingron M: **Introducing Knowledge into Differential Expression Analysis**. *J Comput Biol* 2010, **17**(8):953–967.

12. Bishop CM: *Pattern Recognition and Machine Learning.* New Yorck: Springer Science 2009.

13. Barski A, Jothi R, Cuddapah S, Cui K, Roh T, Schones DE, Zhao K: **Chromatin poises miRNA- and protein-coding genes for expression**. *Genome Res* 2009, **19**(10):1742–1751.
